# Impacts of various amendments on the microbial communities and soil organic carbon of coastal saline–alkali soil in the Yellow River Delta

**DOI:** 10.3389/fmicb.2023.1239855

**Published:** 2023-09-14

**Authors:** Runang Liu, Beijia Liang, Huili Zhao, Ying Zhao

**Affiliations:** College of Resources and Environmental Engineering, Ludong University, Yantai, China

**Keywords:** carbon sequestration, microbial community, straw addition, desulfurization gypsum, saline-alkali soil

## Abstract

The utilization of industrial and agricultural resources, such as desulfurization gypsum and straw, is increasingly favored to improve saline alkali land. However, there is still a lack of comprehensive study on the mechanism of organic carbon turnover under the conditions of desulfurization gypsum and straw application. We studied the changes in soil chemical performance, microbial diversity, and microbial community structure in soils with the addition of various levels of straw (no straw, S_0_; low straw, S_l_; medium straw, S_m_; and high straw, S_h_) and gypsum (no gypsum, DG_0_; low gypsum, DG_l_; and high gypsum, DG_h_) in a 120-day incubation experiment. The bacterial and fungal community richness was higher in the S_m_DG_l_ treatment than in the S_m_DG_0_ treatment. The microbial community evenness showed a similar pattern between the S_m_DG_l_ and S_m_DG_0_ treatments. The combination of the straw and desulfurization gypsum treatments altered the relative abundance of the main bacterial phyla Bacteroidetes and Firmicutes and the dominant fungal class Sordariomycetes, which increased with the enhancement of the SOC ratio. The combination of the straw and desulfurization gypsum treatments, particularly S_m_DG_l_, significantly decreased the soil pH and exchangeable sodium percentage (ESP), while it increased the soil organic carbon, microbial biomass carbon, and activities of soil enzymes. Improvement in the soil salinization environment clearly drove the changes in bacterial α-diversity and community, particularly those in the soil carbon fractions and ESP. In conclusion, these findings provide a strong framework to determine the impact of application practices on soil restoration, and the information gained in this study will help to develop more sustainable and effective integrated strategies for the restoration of saline–alkali soil.

## Introduction

Saline–alkali soil in coastal China covers a large area and is widely distributed. There are obstacles, such as serious salinization, low fertility, poor structure, and low and unstable crop yields (Lv et al., 2015). The Yellow River Delta, with an area of approximately 5 × 10^5^ hm^2^, is a typical coastal saline–alkali soil and a main resource of reserve land. However, the soil as a whole is slightly alkaline with a high salt content and low soil organic matter (SOM) in the topsoil, and the soil requires improvement before cultivation (Lv et al., 2015; Wu et al., 2019; Ou et al., 2020). In the context of the current limitations in land resources and population growth, it is theoretically and practically important to explore measures to improve the fertility of coastal saline–alkali land in this region to ensure the high-quality development of regional agriculture.

The primary purpose and basic requirements of improving saline land are to reduce salinity and improve soil structure and fertility. Therefore, it is equally important to significantly reduce saline–alkali barriers and improve soil fertility. SOM is a key factor that determines soil fertility, and increasing the content of SOM will also help to improve saline–alkali land (Lal, 2016). The content of soil organic carbon (SOC) in farmland is associated with the balance of generation and its loss by mineralization (Xu et al., 2019). Currently, crop straw returning to the field is viewed as the main measure to improve the content of SOC of farmland in China, and it is also a valuable method to improve saline–alkali soil (Zhao et al., 2018a; Zhao H. L. et al., 2019; Jin et al., 2020; Zhu et al., 2021). The process of SOC turnover after the input of exogenous organic materials is controlled by soil microorganisms. However, the special habitat of soil with high salinity in the Yellow River Delta will inhibit microbial activity, and high sodium (Na^+^) will destroy the soil aggregate structure, which affects the effective transformation of exogenous straw carbon into the organic carbon pool (Xie et al., 2017). Therefore, during the process of improving the saline–alkali soil in the study area, it is of great importance to explore the scientific mechanism of methods that could improve the rate of retention of carbon by straw.

Desulfurized gypsum, a byproduct of coal-fired power plants, is basically similar to natural gypsum owing to its main component of calcium sulfate (CaSO_4_·2H_2_O). Thus, it can reduce the damage of alkali by replacing the Na^+^ ions on soil colloids and also improve soil aggregation and microbial activity, which is a topic of major concern (Luo et al., 2018). The combination of straw and desulfurized gypsum has become an important measure to enhance saline–alkali soil. For example, the combined improvement measures of straw and desulfurized gypsum can significantly reduce soil salinity compared with single measures (Liao et al., 2020). It was also found that a single application of straw or desulfurized gypsum could increase SOC content (Zhao Y. G. et al., 2019). In the semi-arid and semi-humid soda saline–alkali soil area of northeastern China, the application of desulfurization gypsum significantly enhances the abundance of arbuscular mycorrhizal fungi, organic carbon content, and aggregate stability in soil microaggregates (Luo et al., 2018). Treatment with an amendment of treated wastewater_bioenergy sorghum_gypsum assembled a distinctive microbial community structure and accumulated the highest content of SOC (7.66 g kg^−1^). However, it also resulted in the lowest content of soil inorganic carbon (6.63 g kg^−1^) (Somenahally et al., 2023). In addition, under the compound improvement measures, desulfurized gypsum changes the physicochemical and biological properties of the soil, which may also reduce the mineralization of “old carbon” and accelerate the dynamic process of the formation of “new carbon,” thus affecting the sequestration of SOC. Currently, to the best of our knowledge, there has been no in-depth study on its internal mechanism. In view of this, this study proposed to thoroughly analyze the microbial mechanism of the combined improvement of straw and desulfurized gypsum on the sequestration of SOC and provide a theoretical foundation for the rational use of amendments to improve the coastal saline–alkali soil.

Incubation experiments were performed to study the influence of straw, desulfurization gypsum, and their interactions on the sequestration of SOC in saline–alkali soil. We hypothesized that the combination of the straw and desulfurization gypsum would enhance microbial diversity, as well as the abundance of r-strategy microbial species, which would facilitate the storage of SOC. We had the following aims: (a) to assess the alteration in soil chemical characteristics after the addition of straw and desulfurization gypsum, (b) to determine the members of the soil microbial community following the addition of combinations of straw and desulfurization gypsum, and (c) to conduct research on the interactions between the soil microbial communities and soil chemical characteristics.

## Materials and methods

### Characterization of soil and exogenous straw and desulfurization gypsum

Soil samples were taken from the topsoil (0–20 cm) at the Dongying Station of Modern Agriculture of Ludong University (Yantai, China) in July 2020 (37.45°N, 118.58°E) in northeastern China. This field had been planted as a rice (*Oryza sativa)* monoculture (a C_3_ crop) for at least 10 years. When the sample was air dried and thoroughly mixed, the visible crop residues were passed through a 2-mm sieve. The soil with a δ^13^C value of −23.21‰ had a sandy loam texture that contained 6% clay, 27% silt, and 67% sand. The soil samples contained 4.03 g kg^−1^ SOC, 0.24 g kg^−1^ total nitrogen, 0.67 g kg^−1^ total phosphorus, 18.41 mg kg^−1^ total potassium, pH (H_2_O) 7.23, 2.61 mS cm^−1^ of electrical conductivity, and 2.10 g kg^−1^ water-soluble sodium, and the experimental soil is considered to be a medium saline–alkali soil (Zhu and He, 1985).

Air-dried straw (a C_4_ plant) obtained from corn (*Zea mays*) near the Dongying Experimental Station of Ludong University (Shandong, China) in July 2020 was dried in a 60°C oven and cut into pieces approximately 2 cm long, which were then mixed into the soil. The total C and N contents of the straw were 456.13 and 6.81 g kg^−1^, respectively (C:N of 66). The δ^13^C value of maize straw was −13.12‰. There were 25.3, 30.04, and 11.56% of cellulose, hemicellulose, and lignin, respectively.

The desulfurization gypsum applied in the experiment came from Shandong Dongying Shengli Power Plant Co., Ltd. (Shandong, China). As a soil conditioner, its main component is CaSO_4_·2H_2_O, which is rich in beneficial mineral elements required by plants, such as sulfur and calcium carbonate (CaCO_3_), which is present at 255 mg kg^−1^. The soil had a pH of 7.23. The levels of heavy metals in the desulfurization gypsum and samples are shown in Supplementary Table S1 and were below the threshold values of Class II of the Chinese National Standard for Soil Environmental Quality (GB 15618-2018).

### Experimental design

This experiment adopted a 4 × 3 factorial design. One factor was the addition of exogenous straw (no straw, S_0_; 4.5 g straw kg^−1^ soil, low straw, S_l_; 12 g straw kg^−1^ soil, medium straw, S_m_; and 22.5 g straw kg^−1^ soil, high straw, S_h_), and the other was addition of desulfurization gypsum (DG) (no gypsum, DG_0_; 13.5 g gypsum kg^−1^ soil, low gypsum, DG_l_; and 36 g gypsum kg^−1^ soil, high gypsum, DG_h_). Each group had three repetitions and was randomized.

Each soil sample (250 g dry weight equivalent) was pre-incubated for 7 days. The soil was evenly mixed with desulfurization gypsum and/or straw and added to a plastic tank (1.0 L). A volume of 5 ml of urea and diammonium phosphate was dissolved in deionized water and added to each plastic tank as a solution (4.4 g L^−1^ N and 2.1 g L^−1^ phosphorus pentoxide [P_2_O_5_]). The added nutrients, which were equivalent to 5 kg of N and 2 kg of P per ton of straw, were designed to augment the nutrients within the straw to achieve the ratios of C:N:P found in the fine fraction of SOM (Kirkby et al., 2011). The tanks were arranged in a random block design and incubated in the dark at 25°C for 120 days. The soil water content was maintained at 60% water holding capacity by periodic weighing of the tanks and the addition of distilled water when necessary.

### Measurement of soil CO_2_ emissions and soil analysis

The emissions of soil CO_2_ were analyzed as described by Zhao et al. (2018b). Soil samples were collected on the 14th and 120th days (considering the effect of straw availability). Previous studies showed that under appropriate temperate and moisture, straw mineralization was fast in approximately 2 weeks, and the mineralization curve became relatively flat after 120 days, indicating straw availability reduced greatly. Three replicate incubation tanks during each destructive sampling were collected in each treatment (3 tanks × 12 treatments × 2 sampling times). A total of 72 soil samples were packed in hard plastic cassettes and taken to the laboratory for further processing. One portion of every fresh sample was stored at −80°C, and the other portion was stored at 4°C for the analyses of soil microbial biomass carbon (SMBC), dissolved organic carbon (DOC), and enzyme activities. The remaining soil was dried and used to test the properties of soil chemicals. The soil pH was determined based on a mixture of soil and water (1:2.5). The amount of DOC was determined as described by Jones et al. (2012) and performed on a TOC analyzer (Shimadzu TOC-500A, Kyoto, Japan). The microbial biomass carbon was detected using the fumigation extraction method as previously described (Kanerva and Smolander, 2007). The β-glucosidase and β-1,4-xylosidase activities were assayed using the fluorescence method as previously described (DeForest, 2009). The extraction of exchangeable Na^+^ was based on ammonium acetate (NH_4_OAc), and its content was measured using inductively coupled plasma atomic emission spectroscopy (ICP-AES), as previously described (Leeman Labs Inc., Hudson, NH, USA). Values for the cation exchange capacity (CEC) were measured based on the barium chloride (BaCl_2_) and NH_4_OAc methods, as previously described. The levels of ESP were calculated as described by Hanson et al. (1999). The contents of the soluble calcium (Ca^2+^), magnesium (Mg^2+^), and sodium (Na^2+^) were measured using a Prodigy-7 ICP-AES (Leeman Labs Inc., Hudson, NH, USA). The sodium adsorption ratio (SAR) was determined by measuring the levels of Na^+^, Ca^2+^, and Mg^2+^ in the extract. After the incubation, a dry-sieving/winnowing procedure was used to remove any partially degraded plant residue. The levels of organic carbon in the sample and straw were calculated using an elemental analyzer (Vario MAX, Elementar, Jena, Germany). The natural abundance of δ^13^C in the straw and SOC was measured as described by Zhao et al. (2017, 2018b). In total, 1 g of soil was preconditioned for 12 h with 10 ml of 1 M HCl to remove the carbonate. We determined the content of straw-derived C, which was a newly formed SOC, as follows:


(1) $$\begin{array}{l} \text{Newly formed SOC}=\;{\text{C}}_{\text{t }}\times \;\frac{{\text{δ}}^{13}{\text{C}}_{\text{t}}-{\text{δ}}^{13}{\text{C}}_{\text{s}}}{{\text{δ}}^{13}{\text{C}}_{\text{straw}}-{\text{δ}}^{13}{\text{C}}_{\text{s}}} \end{array}$$

where δ^13^C_t_ is the δ^13^C of SOC in the amended soil samples following incubation; δ^13^C_s_ is in the primary soil samples, and δ^13^C_straw_ represents the δ^13^C of straw and straw plus the desulfurization gypsum mixture.

The formula for the content of sequestered SOC is shown as follows:


(2) $$\begin{array}{l} \text{Sequestered SOC }=\text{ SO}{\text{C}}_{\text{final}}-\text{SO}{\text{C}}_{\text{before}} \end{array}$$

where SOC_final_ and SOC_before_ are the SOC contents after and before the incubation experiment, respectively.

The soil microbial DNA was extracted from 0.5 samples using a FastDNA SPIN Kit for Soil (MO BIO Laboratories, Inc., Carlsbad, CA, USA), according to the manufacturer's instructions. The DNA was purified using an Ultra-Clean DNA Purification Kit (MO BIO Laboratories, Inc.).

The V_3_-V_4_ regions of the 16S rRNA genes were amplified using primers 338f and 806r with a 12 nt unique barcode at the 5′ end. For fungi, the ITS region of the fungal rRNA gene was amplified using primers ITS1F and ITS2. The purified amplicons obtained from the merger process were paired and sequenced using an Illumina MiSeq PE300 platform (Illumina, San Diego, CA, USA).

### Analysis of the illumina MiSeq sequencing data

QIME 1.8.0 software was used to process the MiSeq data. The quality of analytical results was improved by removing sequences with quality scores < 20 and sequence lengths < 150 bp during quality checks. To prevent bias caused by sequencing depth, 54,927 readings/395 amplified sequence variants (ASVs) of 16S genes and 50,676 readings/60 ASVs of ITS sequences were randomly selected from each sample during the processing process. Statistical analysis was conducted on these samples to determine the microbial diversity and community structure. ASVs evaluated the data based on the level of similarity of the sequences at 100%. When all the sequences had been classified, the ribosome database project (RDP) tool was used to divide the samples into different populations. UCLUST was used to classify the 16S rDNA gene sequences with a threshold of 90%, and the corresponding ITS sequences were classified using UCLUST.

### Statistical analysis

All the data were analyzed based on the DPS 7.05 package. A three-way repeated measures analysis of variance (rmANOVA) was utilized to study the influences of straw-derived materials, level of desulfurization gypsum, sampling times and their interactions on soil chemical properties, enzyme activities, diversity index, and relative abundance (RA) of bacteria and fungi. The treatment means were compared by an LSD at *p* < 0.05. Spearman's rank correlation coefficients between soil performance and diversity in the bacteria and fungi were analyzed using DPS 7.05. In addition, a redundancy analysis (RDA) was used to check various changes in the relation between the outer variables (soil chemical properties) and the composition of microbial communities. Partial least squares path modeling (PLS-PM) was utilized to investigate the possible causal relationships among the chemical properties, associated microbial communities, enzyme activities, and soil carbon responses to different amendments.

## Results

### Soil chemical properties and enzyme activities

**Figure 1:**
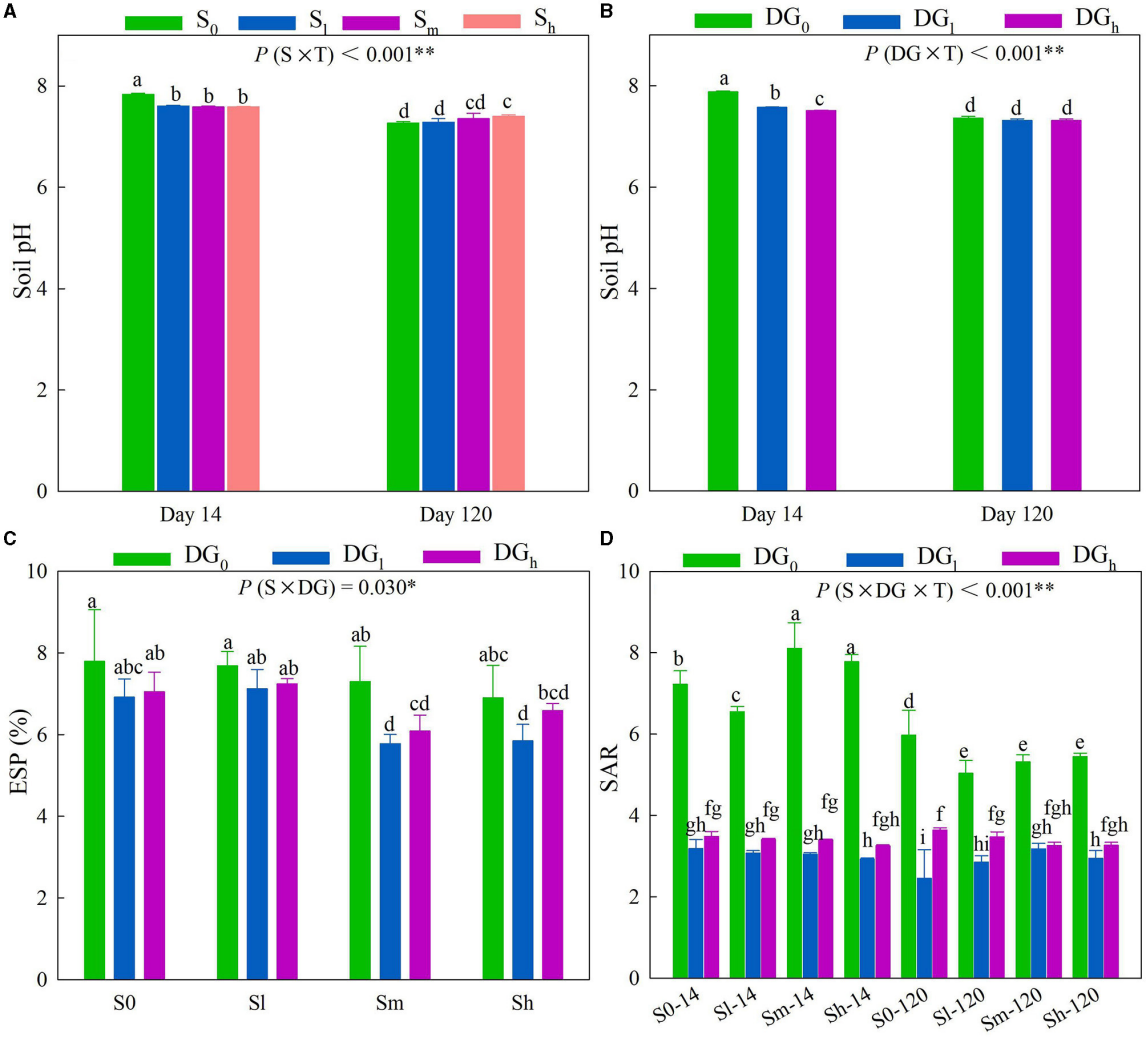
Soil pH **(A, B)**, ESP **(C)**, and SAR **(D)** as influenced by treatment with different levels of straw (no straw, S_0_; 4.5 g straw kg^−1^ soil, S_l_; 12 g straw kg^−1^ soil, S_m_; 22.5 g straw kg^−1^ soil, S_h_) and desulfurization gypsum (no gypsum, DG_0_; 13.5 g gypsum kg^−1^ soil, DG_l_; 36 g gypsum kg^−1^ soil, DG_h_) on days 14 and 120. Different lowercase letters indicate significant differences at the 5% level. The character shows the summary of a rmANOVA with “*p*” values (S, straw level; T, sampling time; DG, Desulfurization gypsum level; and S × T, DG × T, S × DG, S × DG × T, which indicate different interactions). ESP, exchangeable sodium percentage; rmANOVA, repeated measures analysis of variance; SAR, sodium adsorption ratio. *P < 0.05; **P < 0.01.

A significant interaction of S × T and DG × T occurred with respect to the soil pH (Supplementary Table S2) (*P* < 0.01). On day 14, the addition of straw clearly reduced the soil pH, and the soil pH of all the treatments decreased after a period of time (Figure 1A). On day 14, the pH of DG_l_ and DG_h_ treatments was significantly lower than that of the DG_0_ treatment by 0.31 and 0.37 units, respectively, while on day 120, there was no significant difference in the soil pH between treatments (Figure 1B). The interaction of S × DG and S × DG × T significantly affected the ESP and SAR, respectively (Supplementary Table S2) (*P* < 0.05). The ESP of the S_m_DG_l_ treatment was significantly lower than that of the S_m_DG_0_ treatment by 21.62% on the 14th or 120th day (Figure 1C). The SAR of the treatment, in which no gypsum was added, decreased significantly over a period of time. The addition of desulfurization gypsum further decreased the SAR on the 14th and 120th days (Figure 1D). The cumulative CO_2_ emission (C_min − c_) and SMBC were influenced by the interaction of S × DG × T (Supplementary Table S2). The C_min − c_ and SMBC of all the treatments on day 120 increased compared with those on day 14. The addition of gypsum significantly reduced the C_min − c_ in the presence or absence of additional straw (Supplementary Figure S1a). However, on day 120, the combination of treatment with straw and desulfurization gypsum clearly enhanced the content of MBC compared with the addition of only straw (Supplementary Figure S1b). The soil DOC content was affected by the interaction of S × T and DG × T, as well as by the interaction of S × DG (Supplementary Table S2). The addition of straw on days 14 or 120 clearly enhanced the DOC content, while the addition of desulfurization gypsum decreased it (Supplementary Figures S1c, d). In comparison with the S_0_DG_0_, the DOC content increased by 274% in S_m_DG_0_ and 141% in S_m_DG_l_ (Supplementary Figure S1e).

**Figure 2:**
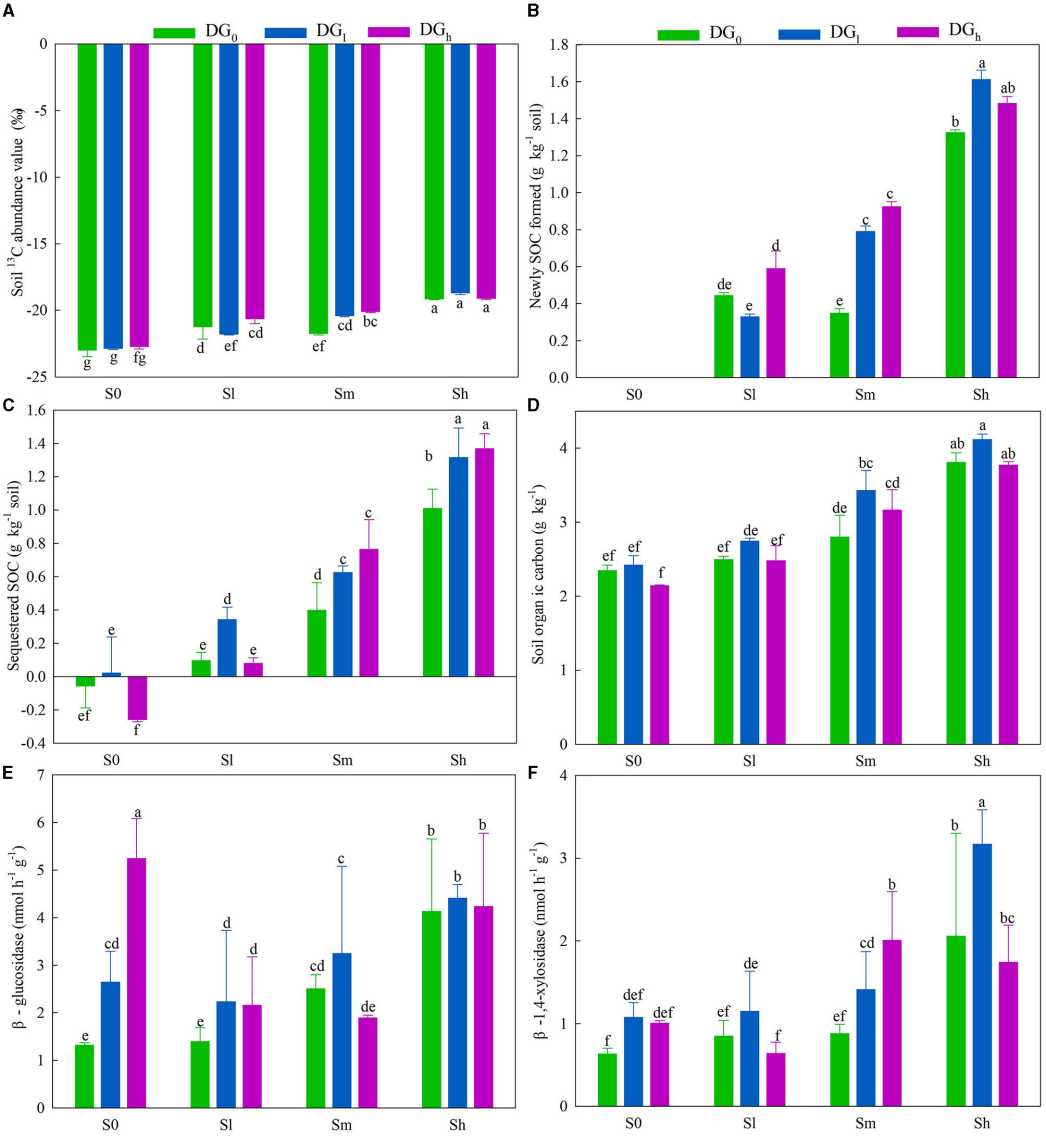
Soil **(A)** ^13^C value, **(B)** newly formed SOC, **(C)** sequestered SOC, **(D)** soil organic carbon, **(E)** β-glucosidase, and **(F)** β-1,4-xylosidase as impacted by various levels of straw (no straw, S_0_; 4.5 g straw kg^−1^ soil, S_l_; 12 g straw kg^−1^ soil, S_m_; and 22.5 g straw kg^−1^ soil, S_h_) and desulfurization gypsum treatments (no gypsum, DG_0_; 13.5 g gypsum kg^−1^ soil, DG_l_; and 36 g gypsum kg^−1^ soil, DG_h_) on day 120. There was no newly formed SOC in the S_0_DG_0_, S_0_DG_l_, and S_0_DG_h_ treatments. DG, desulfurization gypsum; SOC, soil organic carbon.

The application of low, middle, and high amounts of straw increased the value of soil δ^13^C by an average of 7.17, 9.21, and 16.95%, respectively. The desulfurization gypsum increased the value of δ^13^C by 6.90%, on average, when a middle amount of straw was applied (Figure 2A). The application of a high amount of straw increased the newly formed SOC by 198% in the absence of gypsum. On average, the application of desulfurization gypsum increased the formation of new SOC under a medium amount of straw when compared with a lack of gypsum. The application of a low amount of gypsum increased the formation of new SOC by 22.20% when high amounts of straw were applied compared with a lack of gypsum (Figure 2B). The sequestered SOC was significantly affected by the interaction of S × DG (Supplementary Table S2). In comparison with the S_0_DG_0_, the sequestered SOC increased by 0.454 g kg^−1^ in the soil with S_m_DG_0_ and 0.680 g kg^−1^ in the soil with S_m_DG_l_ (Figure 2C). The amount of SOC was significantly affected by the main effects of the application of desulfurization gypsum and straw (Supplementary Table S2). The application of straw increased the SOC content by 35.96% when a middle amount of straw was applied and 69.24% when a high amount of straw was applied compared with the addition of no straw. The SOC content also increased by 11.07% when a low amount of gypsum was applied (Figure 2D). There were significant effects from the application of S × DG on the activities of β-glucosidase and β-1,4-xylosidase (Supplementary Table S2). There was no difference between S_m_DG_0_ and S_m_DG_l_ on the β-glucosidase (Figure 2E). The activity of β-1,4-xylosidase was 1.4-fold higher in soil with S_m_DG_0_ and 2.2-fold higher in soil with S_m_DG_l_ when compared with the S_0_DG_0_ soil (Figure 2F).

### α diversity and β diversity of the soil microbial community

**Figure 3:**
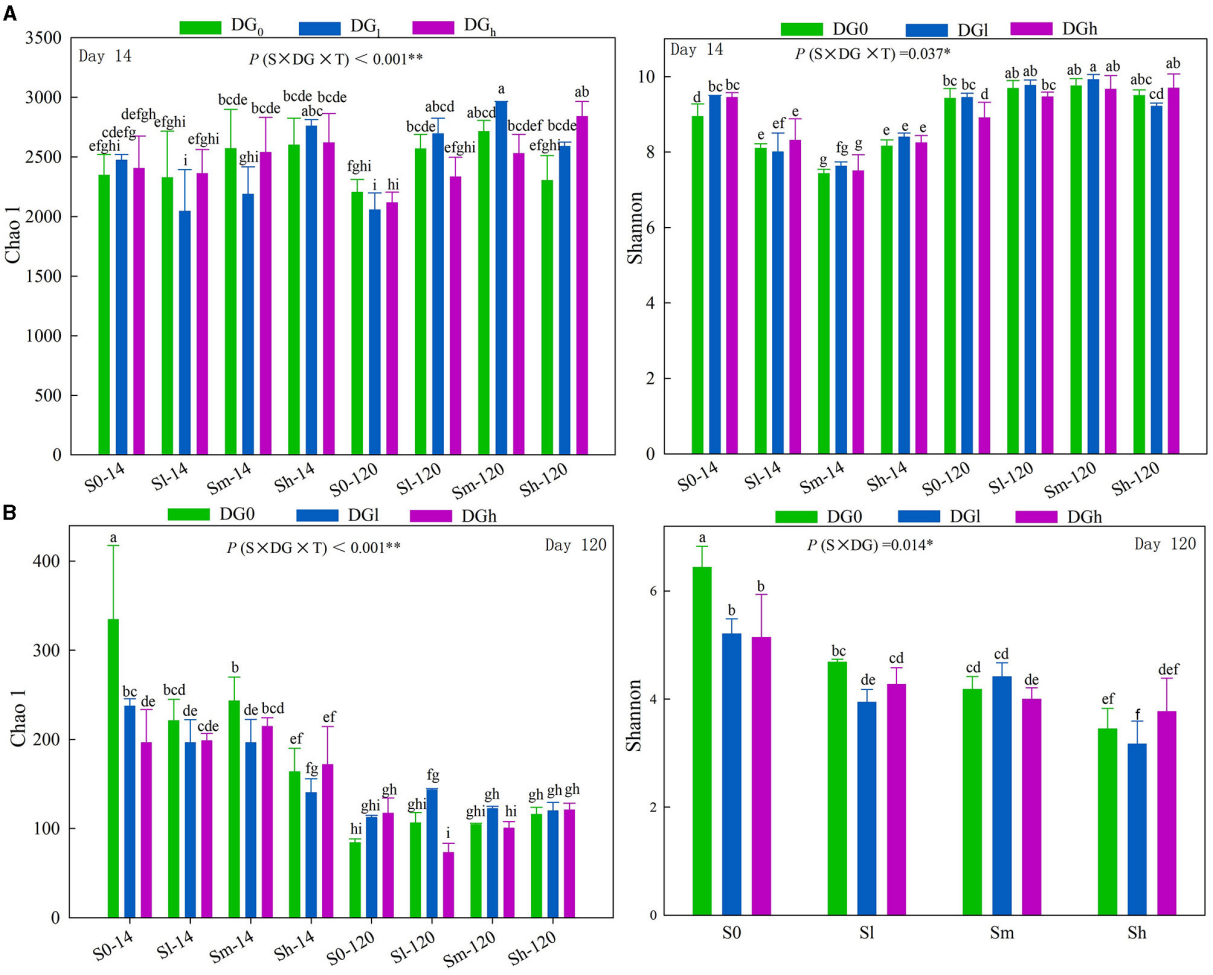
Soil bacterial diversity **(A)** and fungal diversity **(B)** as influenced by different levels of straw and desulfurization gypsum treatments on days 14 and 120. Different lowercase letters indicate significant differences at the 5% level. The character shows the summary of rmANOVA with “*p*” values (S, straw level; T, sampling time; DG, Desulfurization gypsum level; S × T, DG × T, S × DG, S × DG × T, which indicate different interactions). rmANOVA, repeated measures analysis of variance.

The bacterial Chao 1, bacterial Shannon, and fungal Chao 1 indices were significantly affected by the interaction of S × DG × T, and the fungal Shannon index was significantly affected by the interaction of S × DG (Supplementary Table S2). On day 14, only treatment with a high amount of straw increased the Chao 1 index, whereas, on the 120th day, all the treatments of straw addition increased the Chao 1 index compared with the blank treatment. The bacterial Shannon index on the 120th day was higher compared with that on day 14. The Shannon index in the straw treatment decreased on day 14 compared with the blank treatment, while it showed the opposite trend on day 120 (Figure 3A). The fungal Chao 1 index was lower on day 120 than on day 14. The application of desulfurization gypsum also reduced the Shannon index in the blank treatment (Figure 3B).

A principal coordinate analysis (PCoA) showed that the soil bacterial community composition differed significantly between the different straw treatments and desulfurization gypsum (Supplementary Figure S2). The first two axes showed 40.3% of the total difference and the treatment that lacked gypsum differed significantly from the other two treatments (Supplementary Figure S2b). The first two axes can explain 36.5% of the observed fungal community variance (Supplementary Figure S2d). The three treatments of straw addition formed a cohesive group, which was clearly separated from the blank treatments.

### Relative abundance of the richness and diversity of soil microbial taxa

**Figure 4:**
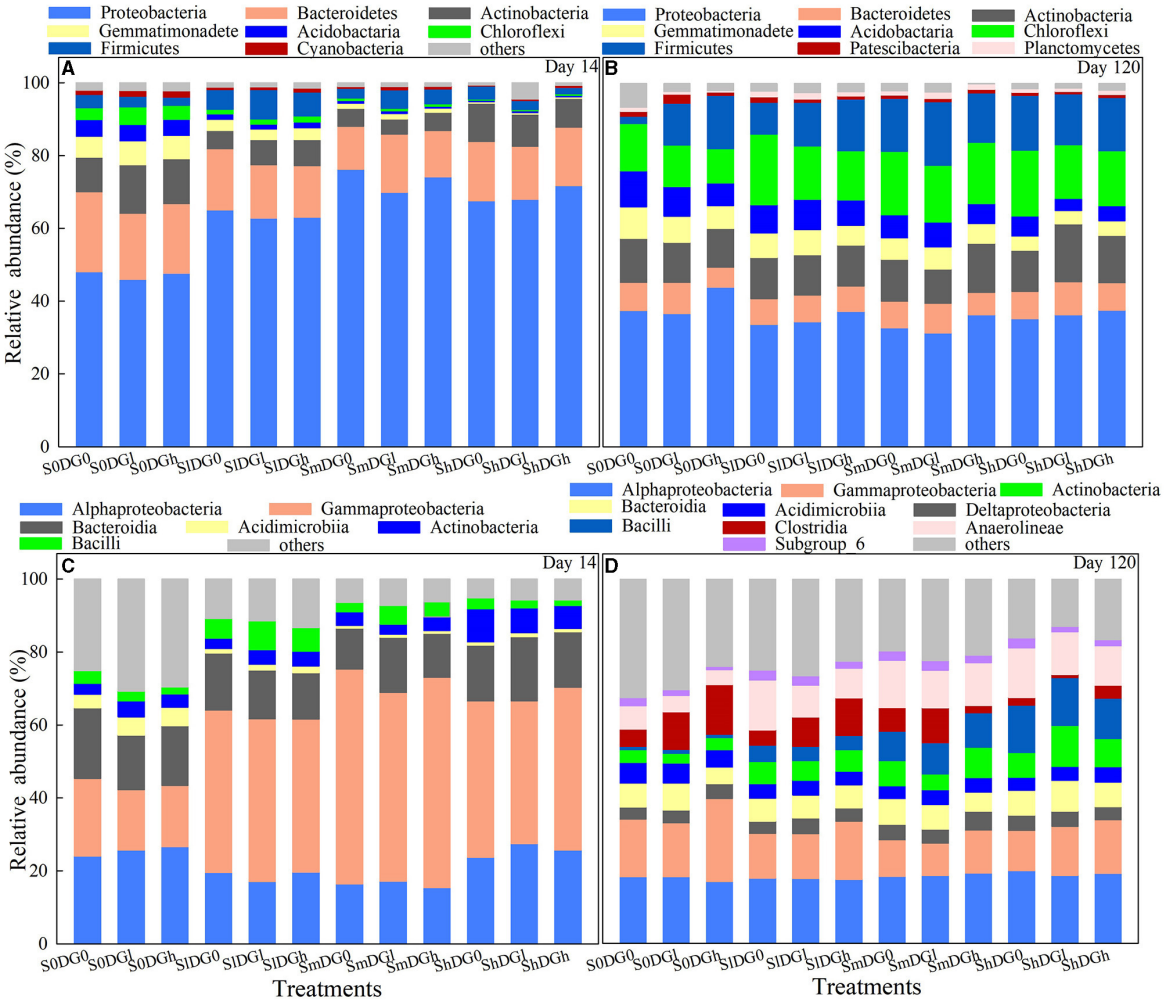
Relative abundance of the main bacterial phyla **(A, B)** and classes **(C, D)** as affected by various levels of straw and desulfurization gypsum treatment (no gypsum, DG_0_; 13.5 g gypsum kg^−1^ soil, DG_l_; and 36 g gypsum kg^−1^ soil, DG_h_).

The effects of straw addition, application of desulfurization gypsum, and their interactions on *Bacteroidetes, Actinobacteria*, and *Chloroflexi* were significant on day 14 (Supplementary Table S3). The application of a low amount of gypsum to the soil reduced the abundance of *Bacteroidetes* in the absence of the straw. The addition of a middle amount of straw further reduced the abundance of *Bacteroidetes*, while the addition of a high amount of straw alleviated the decrease in its abundance (Figure 4A). In the absence of straw application, a low amount of desulfurization gypsum increased the abundance of the *Chloroflexi*. The addition of a low amount of straw reduced the abundance of *Chloroflexi*, while the application of middle and high amounts of straw further decreased the abundance of *Chloroflexi*. The main effects of straw and gypsum clearly affected *Cyanobacteria* (Supplementary Table S3). The addition of straw to the soil decreased the abundance of *Cyanobacteria* by 44.82% when a low amount was added, 51.75% when a middle amount was added, and 74.32% when treated with a high amount compared with the blank treatment (Figure 4A). The application of low and high amounts of desulfurization gypsum increased the abundance of *Cyanobacteria* by 40.88 and 64.08%, respectively.

The relative abundances of *Acidobacteria, Chloroflexi*, and *Firmicutes* were higher on day 120 than on day 14, while those of *Bacteroidetes, Proteobacteria*, and *Gemmatimonadete* showed the opposite trend. *Patescibacteria* and *Planctomycetes* appeared on day 120 (Figure 4B). The interaction between the addition of straw and the application of desulfurization gypsum had an impact on *Actinobacteria, Acidobacteria, Patescibacteria*, and *Planctomycetes* on day 120 (Supplementary Table S3). There was a significantly higher abundance of *Actinobacteria* when there was a combined application of a high amount of straw and a low amount of gypsum compared with the other treatments. The abundance of *Acidobacteria* was also reduced when low and middle amounts of straw were added except for the combined application of a low amount of straw and gypsum. However, treatment with a high amount of straw further reduced the abundance of *Acidobacteria*. In comparison with the S_0_DG_0_, the increase in the abundance of *Bacteroidetes* was −4.9% in S_m_DG_0_ and 5.7% in S_m_DG_l_. In comparison with the S_0_DG_0_, the abundance of *Firmicutes* increased by 154% in S_m_DG_0_ and 176% in S_m_DG_l_. In comparison with the S_0_DG_0_, the abundance of *Planctomycetes* increased by 3.8% in S_m_DG_0_ and 56.2% in S_m_DG_l_ (Figure 4B).

The relative abundances of *Gammaproteobacteria* and *Bacteroidia* were lower on day 120 than on day 14, while *Acidimicrobiia* and *Actinobacteria* showed the opposite trends (Figures 4C, D). *Anaerolineae*, Clostridia, Subgroup_6, and *Deltaproteobacteria* were only relatively abundant on day 120. The abundances of *Anaerolineae, Bacilli*, and *Clostridia* were remarkably distinct between the treatments of added straw and desulfurization gypsum and the blank treatment. These results demonstrate that amendments changed the relative abundance of bacteria.

**Figure 5:**
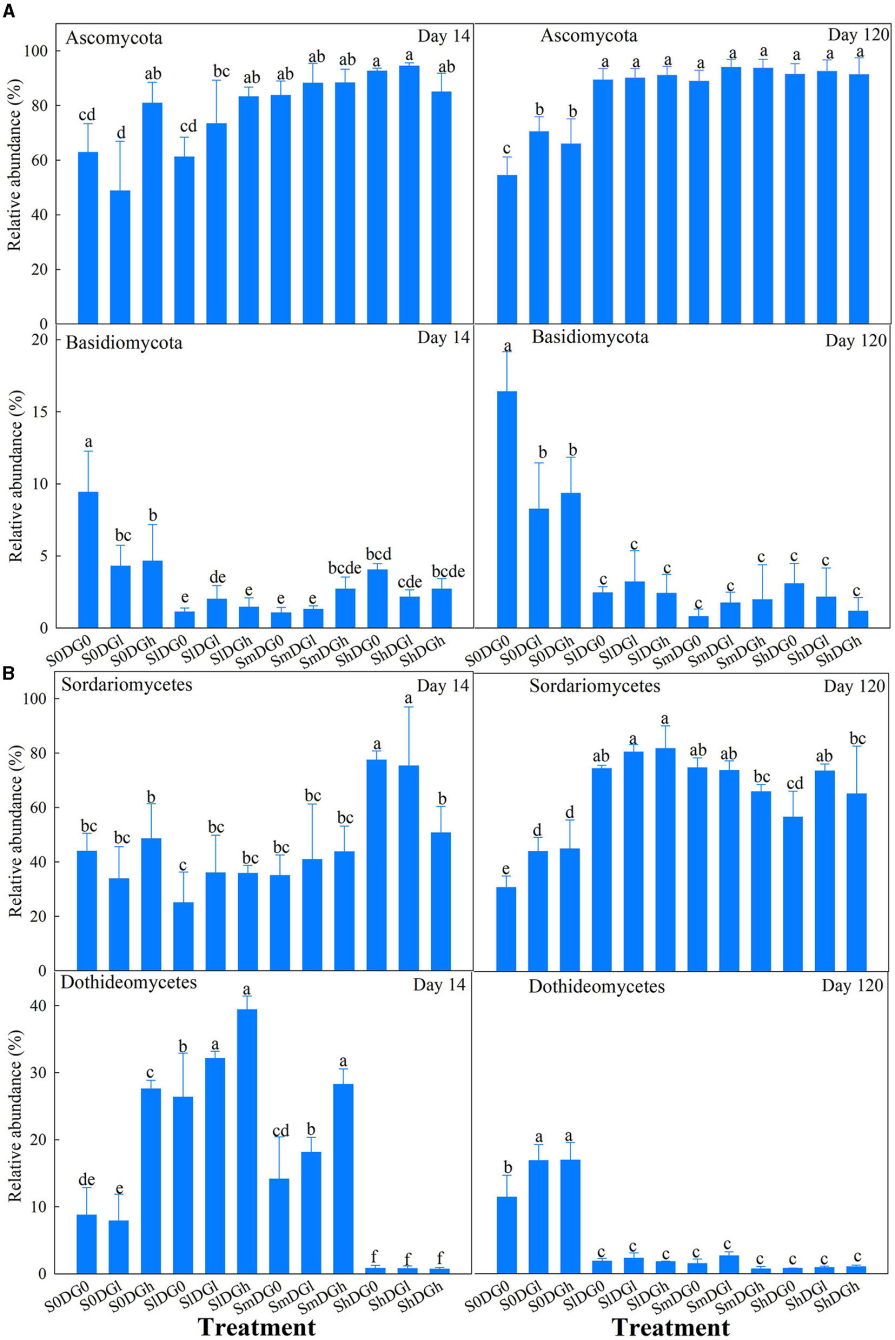
Relative abundance of the main fungal phyla (%) **(A)** and fungal classes (%) **(B)** as affected by different levels of straw and desulfurization gypsum treatments (no gypsum, DG_0_; 13.5 g gypsum kg^−1^ soil, DG_l_; and 36 g gypsum kg^−1^ soil, DG_h_). Different lowercase letters indicate significant differences at the 5% level.

On day 14, the abundances of *Ascomycota* and *Basidiomycota* were clearly impacted by the interaction between the addition of straw and the application of desulfurization gypsum (Figure 5A). The abundance of *Ascomycota* also increased by 40.76% in the absence of additional straw, and with the addition of three levels of straw, the addition of gypsum clearly improved the abundance of *Ascomycota*. On day 120, low and high applications of gypsum also resulted in 29.41 and 21.32% increases in the abundance of *Ascomycota*, respectively, when compared with the blank treatment in the absence of straw. All the treatments at the straw level improved the abundance of *Ascomycota*. On day 14, straw and a low application of gypsum significantly reduced the abundance of *Basidiomycota*. *Basidiomycota* was 79.23, 88.46, and 83.85% less abundant in the low, middle, and high straw treatments on day 120 than in the blank treatment (Figure 5A).

The main effects of the addition of straw and application of desulfurization gypsum significantly affected *Sordariomycetes*. The abundance of *Sordariomycetes* increased by 14.9% when a low amount of gypsum was applied compared with the blank treatment. The application of low, middle, and high amounts of straw increased the abundance of Sordariomycetes by 97.5, 78.9, and 62.9%, respectively (Figure 5B).

### Relationships between microbial diversity and organic carbon fractions and soil enzyme activities

The Chao1 index was positively associated with C_min − c_ and MBC, and the Shannon index was inversely associated with C_min − c_ and DOC on day 7. The Chao1 index was strongly positively associated with C_min − c_, pH, and sequestered SOC on day 120 (Supplementary Figure S3). For the fungal diversity, the Shannon indices were inversely associated with C_min − c_, MBC, DOC, pH, newly formed SOC, and sequestered SOC (Supplementary Figure S4).

### Relationships between the microbial community structure, organic carbon fractions, and soil enzyme activities

**Figure 6:**
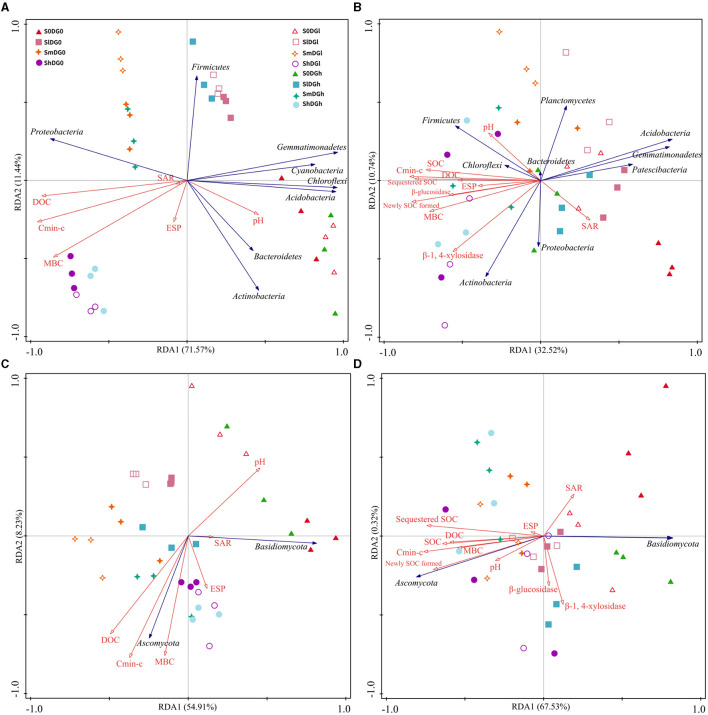
RDA results for enzyme activity, soil properties, and the bacterial community on day 14 **(A)** and day 120 **(B)** and the fungal community on day 14 **(C)** and day 120 **(D)**. C_min − c_, cumulative CO_2_ emission. SOC, soil organic carbon; DOC, dissolved organic carbon; MBC, microbial biomass carbon; ESP, exchangeable sodium percentage; SAR, sodium adsorption ratio; RDA, redundancy analysis.

The selected soil characteristics accounted for 83.01 and 43.26% of the bacterial community changes on the 7th and 120th days, respectively, while those of the fungal community were 63.14 and 67.85%, respectively (Figure 6). The bacterial communities found in the plots with different levels of straw or gypsum formed a group that was separated from the straw- or gypsum-free group along the first axis on the 7th and 120th days (Figures 6A, B). On the 120th day, the abundances of *Actinobacteria, Gemmatimonadetes, Acidobacteria, Chloroflexi, Firmicutes, Patescibacteria*, and *Planctomycetes* were related to soil performance, and the negative correlation indices were as follows: C_min − c_, MBC, DOC, SOC, newly formed SOC, sequestered SOC, and β-1,4-xylosidase (*Gemmatimonadetes* and *Acidobacteria*); C_min − c_, pH, and newly formed SOC (*Patescibacteria*); and β-1,4-xylosidase (*Planctomycetes*). The positively correlated indices included MBC, newly formed SOC, and sequestered SOC (*Actinobacteria*); C_min − c_ and DOC (*Chloroflexi*); and C_min − c_ and β-glucosidase (*Firmicutes*) (Figure 6B, Supplementary Figure S5). There was clearly a positive association between the abundance of *Ascomycota* and C_min − c_, DOC, SOC, newly formed SOC, and sequestered SOC on day 120, and a clear negative association was observed between the abundance of *Basidiomycota* and C_min − c_, SOC, newly formed SOC, and sequestered SOC (Supplementary Figure S6).

**Figure 7:**
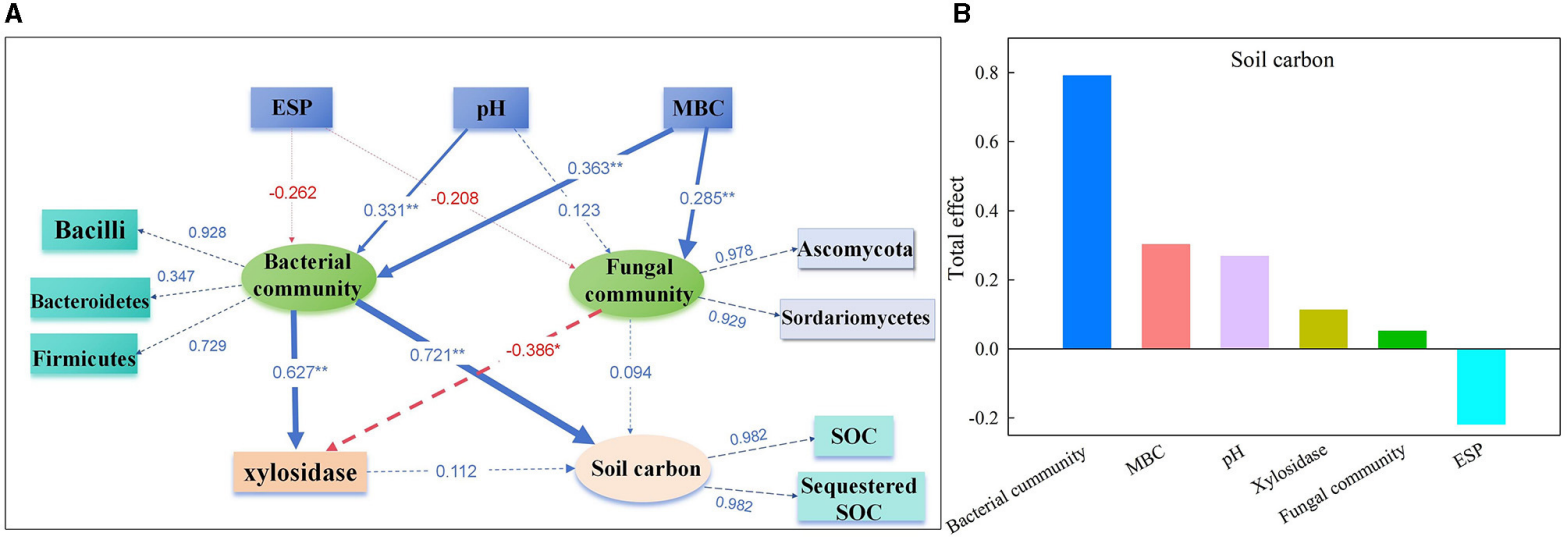
The direct and indirect effects of soil chemical properties, soil microbial communities, and enzyme activities on soil carbon were determined by partial least squares path modeling **(A)**, and the total effects of each parameter on soil carbon **(B)** on day 120. The numbers on the arrows are the standardized path coefficients, and the value of path coefficients is indicated by the width of the arrows. Blue arrows indicate a positive effect. Red arrows indicate a negative effect. The path coefficients and coefficients of determination (R^2^) were calculated after 999 bootstraps. *P < 0.05. **P < 0.01. R^2^, variance explained by the model. The model was evaluated using goodness-of-fit tests, which measure the overall prediction performance.

The PLS-PM identified direct and indirect effects of the soil chemical properties (ESP, pH, and MBC), bacterial community (*Bacilli, Bacteroidetes*, and *Firmicutes*), fungal community (*Ascomycota* and *Sordariomycetes*), and β-1,4-xylosidase activity on the soil carbon (SOC and sequestered SOC) (Figure 7). The soil pH had a significant direct positive relationship with the bacterial community (0.331), and the MBC had a significant positive relationship with the bacterial (0.363) and fungal communities (0.285). In addition, the bacterial community had a direct significant positive relationship (0.627) with the activity of β-1,4-xylosidase and a positive relationship (0.721) with soil carbon. Notably, the fungal community had a significant direct negative impact on the activity of β-1,4-xylosidase (−0.386). The total effects of the bacterial community on soil carbon were 79.1%, followed by MBC (30.2%), pH (26.8%), β-1,4-xylosidase activity (11.2%), fungal community (5.1%), and ESP (−21.8%) (Figure 7).

## Discussion

### Soil biochemical properties

The ESP value under the S_m_DG_0_ treatment was lower than that under S_0_DG_0_ (Figure 1C). The S_m_DG_l_ treatment clearly reduced the ESP compared with the S_0_DG_0_. Desulfurization gypsum is a type of typical soil conditioner, and it can release Ca^2+^ into the soil (Schultz et al., 2017). The increase in Ca^2+^ effectively reduces the concentrations of carbonate (CO${}_{3}^{2-}$) and bicarbonate (HCO${}_{3}^{-}$), thus causing lower values of pH and ESP in the soil (Figure 1) (Chi et al., 2012; Zhao et al., 2020). A higher content of Ca^2+^ can reduce the values of ESP and SAR through the “valence dilution” manner. Xie et al. (2017) found that the special habitat of saline–alkali soil with the high salinity in the Yellow River Delta will inhibit microbial activity, and high contents of Na^+^ will destroy the soil aggregate structure, which reduces the effective sequestration and transformation of exogenous straw carbon. The addition of organic materials is necessary to improve saline–alkali soil. However, Ca^2+^ in the chemical amendments can combine soil minerals and organic substances and thus physically protect the SOC. Similarly, there was 6.24-fold more highly sequestered SOC under S_m_DG_l_ and 3.97-fold under S_m_DG_0_ compared with that under S_0_DG_0_ in this study (Figure 2C), which supports the hypothesis described above. Similar trends were found for the SOC content (Figure 2D). This trend was also consistent with the differences in C_min − c_ among the treatments (Supplementary Figure S1), and the distinction might be owing to the formation of calcium-bonded organic carbon after desulfurization gypsum had been added to the soil. The addition of desulfurization gypsum could enhance the stability of SOC and reduce the conversion of the SOC to CO_2_. Similarly, Kim et al. (2017) found that in coastal tideland soils, the combination of straw return and gypsum application was more effective at enhancing the stability of aggregates compared with the addition of rice straw alone. The reason was that the Ca^2+^ in gypsum replaced the exchangeable Na^+^ on soil colloids and promoted the flocculation of soil clay particles. Moreover, the addition of gypsum to straw-returning soil reduced the DOC content (Supplementary Figure S1), which is consistent with the previous findings by Ehsan et al. (2021). On the one hand, Ca^2+^ helps to stabilize organic matter and increases the adsorption of calcium (Tavakkoli et al., 2015), thereby reducing the DOC content. Alternatively, our research showed that the DOC content decreased with the low pH under the gypsum treatments. The soil pH changes the stability of SOM by affecting the dissolution of soluble organic matter and the organic-mineral combination, thus affecting its degradation by soil microorganisms (Rath and Rousk, 2015). A study by Ehsan et al. (2021) provides evidence for this hypothesis and showed that the application of gypsum continuously reduced the soil pH, dissolved the organic C, and resulted in higher organic C than the unmodified control. Soil microbial biomass carbon (SMBC) can indirectly reflect soil microbial activity. The highest level of SMBC appeared in the combination of straw and gypsum treatments, followed by straw treatments. This result could be primarily attributed to the improvement in the soil salinization environment with significant reductions in pH, ESP, and SAR (Figure 1), which improved the efficiency of microorganisms to utilize the soil exogenous carbon and effectively improved the soil MBC content (Heng et al., 2023) (Supplementary Figure S1).

In this study, it is worth noting that the treatment of desulfurization gypsum reduced the DOC and enhanced the contents of SOC and MBC. In addition, a reduction in the soil pH, ESP, and SAR will enhance the physical performance of the soil, which leads to a change in the activity and structure of the microbial community, thus affecting the turnover process of the SOC.

### Soil microbial species richness and diversity

Two important indicators of microbial diversity—the Shannon and Chao1 indices—represent the complication and stabilization of the microbial community (Wagg et al., 2019), which are important signs of soil health (Bender et al., 2016; Vergani et al., 2017). The former reflects the abundance of species, while the latter reflects the richness of species. This study found that the soil bacterial diversity was enhanced by the addition of straw and desulfurization gypsum on day 120 (Figure 3A). This may be significant since greater diversity may enhance the resistance of microorganisms to interference and has beneficial effects on soil productivity by inhibiting diseases (Sapp et al., 2015). The abundance and diversity of soil microorganisms are mostly associated with pH and soil nutrition (Maarastawi et al., 2018). The addition of desulfurization gypsum can reduce salinity and facilitate the decomposition of external organic material. This introduces more organic matter, which results in a high diversity of microbial communities. Therefore, the activities of enzymes increase. In addition, the Chao 1 index significantly positively correlated with the C_min − c_ and sequestered SOC on day 120 (Supplementary Figure S1). Thus, the influence of the quantity of SOM on bacterial diversity and richness cannot be ignored. Straw return offers a C source for microbes, which enables their diversity (Maarastawi et al., 2018). The additional nutrient input provided by the straw may cause the higher uniformity and richness observed in the straw treatment (Chen et al., 2017). Some researchers have studied the effects of long-term straw return on soil bacteria from different perspectives. The impact of diversity has achieved a series of results, but owing to various factors, no consensus has currently been reached. Bu et al. (2020) found that returning straw to the field has some ability to improve the soil structure while also increasing the bacteria α diversity in acidic soil, but it does not have an impact on alkaline soil. The reasons for this are not yet clear, and an in-depth analysis is needed to more effectively meet the treatment requirements related to straw returning to the field (Zhang H. L. et al., 2021).

Higher biodiversity of fungi in the soil results in a more stable system (Chaer et al., 2009). In this study, the mixture of straw and desulfurization gypsum caused a significant increase in fungal species richness when compared with the treatments of just straw on day 120 (Figure 3B), which indicates that the application of gypsum substantially increased the degree of fungal α-diversity and caused changes in the composition of the fungal community. The RDA and PLS-SM results indicate that the pH is not the prominent factor in the formation of the fungal community (Figures 6D, 7). Similar to our findings, Chaer et al. (2009) found that the diversity of the soil fungal community in the entire black soil area was primarily determined by the level of total carbon in the soil rather than its pH. The Shannon index for soil fungi had a clear inverse association with MBC, SOC, newly formed SOC, and sequestered SOC on day 120 (Supplementary Figure S2). Clearly, this low-quality residue is only favored by those few fungal species that could utilize such a residue, while the overall uniformity of fungi decreased again (Marschner et al., 2003). Thus, although applying crop residues can enhance the abundance of microorganisms, it indeed reduced the diversity of microorganisms (fungi) in our case. On the 14th day, it was observed that the fungal Chao 1 and Shannon indices clearly reduced the decrease in pH values (Supplementary Figure S2), which was very similar to the findings by Wang et al. (2015). As the pH value decreased, some tolerable taxa became scarce or even disappeared, and the previous habitat taxa balance was broken. This resulted in lower fungal community diversity.

### Relative abundance of soil microbial taxa

The soil microbiome exerts vital functions in element cycles and the stability of soil composition (Daynes et al., 2013) owing to the mineralization of various organic substances that enhance its activity. Reportedly, 20 bacterial strains of *Firmicutes* and *Bacteroidetes* were isolated from saline–alkaline soils in northeastern China (Shi et al., 2012), which is consistent with the current research findings. After 120 days of decomposition, the abundances of *Chloroflexi, Acidobacteria, Actinobacteria*, and *Gemmatimonadetes* belonged to the oligotrophic (or *K*-selected) group (Di Lonardo et al., 2017), which was consistent with their significant advantages in the late stages that were identified in other research (Dilly et al., 2004; Moitinho et al., 2018). The abundances of *Bacteroidetes* and *Firmicutes* clearly positively associated with the levels of soil nutrients, while the abundances of *Gemmatimonadetes* and *Acidobacteria* displayed an inverse correlation with SOC and sequestered SOC on day 120 (Figure 6B, r = 0.48, *P* = 0.018 and r = 0.48, *P* = 0.018, respectively). The addition of straw and desulfurization gypsum more clearly enhanced the amount of SOC than that added separately (Figure 2D), which is consistent with the addition of straw and desulfurization gypsum that promotes the growth of *Bacteroidetes* and *Firmicutes* (Figure 6A). *Planctomycetes* can be used to participate in the soil C and N cycles (Mori and Kamagata, 2014). Treatment with straw and desulfurization gypsum can supply a large number of C and N sources, which led to a higher abundance of *Planctomycetes* on day 120 (Figure 6B). A structural equation model revealed that bacterial communities associated with ESP, pH, and MBC indirectly affected soil carbon by changing β-1,4-xylosidase activity and community composition under the addition of amendments (Figure 7). Some research studies have shown that *Bacteroidetes* and *Firmicutes* can produce hydrolases (including β-1,4-xylosidase and β-glucosidase) to break down plant biomass (Rao et al., 2021). Previous reports classified *Bacteroidetes* and *Firmicutes* as copiotrophs (Tang et al., 2022), which use labile forms of C for growth and metabolism and grow faster in a nutrient-rich environment.

Fungi are very important in soil ecosystems since they participate in a series of ecological functions, which also include promoting the processes of N and C cycles (Anderson and Cairney, 2004). The data showed that Ascomycota was the most abundant phylum and was followed by Basidiomycota (Figure 7), which indicated that it can withstand stress conditions. Moreover, the abundance of Ascomycota was higher on day 120 when straw and gypsum were added than the treatments that lacked both amendments, which can promote the soil carbon cycle and the absorption of N by plants. Another phylum Basidiomycota negatively correlated with the SOC fractions on day 120 (Supplementary Figure S4). Owing to the rapid metabolism of nutrients in the mycelia of this phylum, its abundance is associated with the degradation of straw residue (Freedman et al., 2015). From a functional perspective, it is widely hypothesized that the degradation of lignocellulose is associated with Basidiomycota, which could explain the relatively high frequency of this group in the late stage. Basidiomycota can be regarded as K-strategists because they appear late in the degradation stage and can degrade recalcitrant organic matter. The abundance of Basidiomycota decreased following all the treatments of applying straw or gypsum. Ascomycetes and Basidiomycota are key decomposers in the carbon cycle and can secrete digestive enzymes to transform organic substances into smaller molecules. The members of Ascomycota are the main decomposers in agricultural soils. *Sordariomycetes* and *Dothideomycetes* were the two main fungal classes in the sample (Figure 7). *Sordariomycetes* were relatively more abundant on day 120 under the addition of desulfurization gypsum and straw when no treatments were added (Figure 7). This class can produce cellulases and promote the decomposition of straw (Phosri et al., 2012). In this study of Ascomycota fungi, the second most abundant numbers were members of *Dothideomycetes* (Figure 7), which are related to cell wall decay (Freedman et al., 2015). As saprophytes and degradants of plant biomass, they are important for the global carbon cycle (Goodwin and Kema, 2009). This class was enhanced in the DG group on day 120, which indicated that the treatment with desulfurization gypsum can enhance the soil properties and increase the contributions of microorganisms to the decomposition of litter. However, *Dothideomycetes* had a clear inverse correlation with the carbon fractions on day 120 (Supplementary Figure S5), and the addition of straw reduced the abundance of this class. It is associated with higher carbon fractions under straw addition treatments, which is consistent with the findings by Zhang M. M. et al. (2021) in research on the soils of the Loess Plateau in China.

### Predicted function of the bacterial community

We used PICRUST2 for the functional prediction of the Kyoto Encyclopedia of Genes and Genomes (KEGG) against the SILVA annotation of the 16S sequences (Supplementary Figure S6). Statistical analysis revealed changes in the predicted function of the microbiome in soils amended with straw and desulfurization gypsum. In the soil, the addition of desulfurization gypsum changed the functions involved in carbon metabolism, including carbohydrate metabolism, arginine and proline metabolism, and starch and sucrose metabolism, among others, and the effects were related to the levels of exogenous substances.

## Conclusion

The addition of straw and desulfurization gypsum dramatically changed the community structure of microbes. The abundances of *Bacteroidetes, Firmicutes*, and *Sordariomycetes* were positively associated with the level of SOC, which indicated that the increase in SOC may appear with changes in the abundance of bacterial flora. Improvement in the soil saline environment promoted remarkable changes in the bacterial community, particularly those in the soil pH, ESP, and MBC. In total, the effects of soil ameliorants on SOC and the microbial community varied depending on the changes in soil chemical performance caused by the addition of amendments. These results provide information to recognize the correlations between the microbial community and the restoration of soil in response to various amendments and insight into the restoration of saline–alkali soil.

## Data availability statement

The datasets presented in this study can be found in online repositories. The names of the repository/repositories and accession number(s) can be found in the article/Supplementary material.

## Author contributions

RL: concept, review, writing, and editing. BL: methodology, review, writing, and editing. HZ: investigation, data management, and fund acquisition. YZ: supervise project management. All authors have participated in this article and approved the submitted version.
