# Supplementary material for: Impacts of various amendments on the microbial communities and soil organic carbon of coastal saline–alkali soil in the Yellow River Delta
Source: Front Microbiol. 2023 Sep 14;14:1239855. doi: 10.3389/fmicb.2023.1239855 (PMC10539599; doi:10.3389/fmicb.2023.1239855)
Supplement: Supplementary file 1 [file Data_Sheet_1.docx]

**TABLE S1** Heavy metals in soil and gypsum (mg kg^-1^).

| Materials |  | C_r_ | P_b_ | C_d_ | H_g_ | A_s_ |
| --- | --- | --- | --- | --- | --- | --- |
| Desulfurization gypsum |  | 13.16±0.28 | 1.48±0.06 | 0.01±0.001 | 2.16±0.18 | - |
| Soil |  | 24.08±0.32 | 10.01±0.61 | 0.15±0.03 | 0.03±0.003 | 7.75±0.15 |
| GB15618-2018 Class II standard (pH>7.5) |  | 250 | 170 | 0.60 | 3.40 | 25.00 |
| GB15618-2018 Class Ⅰ standard (Natural background) |  | 90.00 | 35.00 | - | 0.15 | 15.00 |

**TABLE S2** Summary (*p* value) of three-way repeated measures analysis of variance of the effects of straw residue, desulfurization gypsum, sampling time, and their interactions on soil chemical characteristics, enzyme activity and microbial diversity.

| Source | Straw (S) | Gypsum (DG) | Time (T) | S × T | DG × T | S × DG | S× DG×T |
| --- | --- | --- | --- | --- | --- | --- | --- |
| pH | <0.001** | <0.001** | <0.001** | <0.001** | <0.001** | 0.188 | 0.289 |
| ESP | <0.001** | <0.001** | 0.020* | 0.998 | 0.999 | 0.030* | 0.999 |
| SAR | 0.006** | <0.001** | <0.001** | 0.179 | <0.001** | <0.001** | <0.001** |
| Cmin-c | <0.001** | <0.001** | <0.001** | <0.001** | <0.001** | <0.001** | 0.004** |
| MBC | <0.001** | <0.001** | <0.001** | <0.001** | <0.001** | <0.001** | <0.001** |
| DOC | <0.001** | <0.001** | 0.022* | <0.001** | <0.001** | <0.001** | 0.745 |
| Soil organic carbon | <0.001** | 0.022* | - | - | - | 0.660 | - |
| Newly SOC formed | <0.001** | <0.001** | - | - | - | 0.004** | - |
| Sequestered SOC | <0.001** | <0.001** | - | - | - | 0.004** | - |
| β-glucosidase | <0.001** | <0.001** | - | - | - | <0.001** | - |
| β-1,4-xylosidase | <0.001** | <0.001** | - | - | - | <0.001** | - |
| Bacterial Chao 1 | <0.001** | 0.955 | 0.242 | <0.001** | 0.086 | 0.256 | 0.004^**^ |
| Bacterial Shannon | <0.001** | 0.353 | <0.001** | <0.001** | 0.053 | 0.452 | 0.037^*^ |
| Fungal Chao 1 | <0.001** | 0.009^**^ | <0.001** | <0.001** | <0.001** | 0.052 | <0.001** |
| Fungal Shannon | <0.001** | 0.003^**^ | 0.055 | 0.756 | 0.068 | 0.002^**^ | 0.816 |

Note: C_min-c_, cumulative CO_2_-C; MBC, microbial biomass carbon; DOC, dissolved organic carbon; ESP, exchangeable sodium percentage; SAR, sodium adsorption ratio. **P* < 0.05, ***P* < 0.01. -, indicates that it does not exist.

**TABLE S3** Analysis of variance of the effects of different levels of straw and desulfurization gypsum addition, as well as their interactions, on the relative abundance of the dominant bacterial phyla (relative abundance >1%) at days 14 and 120 of the incubation.

| Incubation time | Phylum | Different amount of straw(S) | |  | Desulfurization gypsum(DG) | |  | S×DG | |
| --- | --- | --- | --- | --- | --- | --- | --- | --- | --- |
|  |  | *F* | *P* |  | *F* | *P* |  | *F* | *P* |
| Day 14 | Proteobacteria | 120.10 | <0.001 |  | 2.62 | 0.094 |  | 0.99 | 0.449 |
|  | Bacteroidetes | 19.22 | <0.001 |  | 1.95 | 0.165 |  | 3.12 | 0.021 |
|  | Actinobacteria | 148.47 | <0.001 |  | 3.89 | 0.034 |  | 11.39 | <0.001 |
|  | Gemmatimonadete | 257.65 | <0.001 |  | 0.59 | 0.564 |  | 0.92 | 0.500 |
|  | Acidobactaria | 252.31 | <0.001 |  | 0.29 | 0.748 |  | 0.20 | 0.974 |
|  | Chloroflexi | 157.24 | <0.001 |  | 4.28 | 0.026 |  | 3.04 | 0.024 |
|  | Firmicutes | 15.88 | <0.001 |  | 1.75 | 0.195 |  | 1.80 | 0.142 |
|  | Cyanobacteria | 50.03 | <0.001 |  | 13.03 | <0.001 |  | 1.13 | 0.375 |
| Day 120 | Proteobacteria | 4.73 | 0.009 |  | 5.50 | 0.011 |  | 0.48 | 0.82 |
|  | Bacteroidetes | 1.09 | 0.371 |  | 5.57 | 0.010 |  | 1.05 | 0.417 |
|  | Actinobacteria | 2.73 | 0.066 |  | 0.23 | 0.794 |  | 2.75 | 0.035 |
|  | Gemmatimonadete | 22.65 | <0.001 |  | 3.73 | 0.039 |  | 1.44 | 0.241 |
|  | Acidobactaria | 26.49 | <0.001 |  | 8.51 | 0.002 |  | 2.77 | 0.034 |
|  | Chloroflexi | 16.03 | <0.001 |  | 11.16 | <0.001 |  | 1.28 | 0.304 |
|  | Firmicutes | 2.10 | 0.126 |  | 1.86 | 0.177 |  | 1.04 | 0.426 |
|  | Patescibacteria | 11.65 | <0.001 |  | 4.43 | 0.023 |  | 6.55 | <0.001 |
|  | Planctomycetes | 9.66 | <0.001 |  | 1.07 | 0.361 |  | 2.56 | 0.046 |

Note: **P* < 0.05, ***P* < 0.01.

**TABLE S4** Analysis of variance of the effects of different levels of straw and desulfurization gypsum addition, as well as their interactions, on the relative abundance of the dominant bacterial class (relative abundance >0.8%) at days 14 and 120 of the incubation.

| Incubation time | Class | Different amount of straw(S) | |  | Desulfurization gypsum(DG) | |  | S×DG | |
| --- | --- | --- | --- | --- | --- | --- | --- | --- | --- |
|  |  | *F* | *P* |  | *F* | *P* |  | *F* | *P* |
| Day 14 | Alphaproteobacteria | 51.01 | <0.001 |  | 0.85 | 0.440 |  | 1.75 | 0.155 |
|  | Gammaproteobacteria | 106.09 | <0.001 |  | 2.16 | 0.138 |  | 0.78 | 0.589 |
|  | Bacteroidia | 10.61 | <0.001 |  | 2.02 | 0.156 |  | 3.75 | 0.010 |
|  | Acidimicrobiia | 299.56 | <0.001 |  | 10.29 | <0.001 |  | 3.97 | <0.001 |
|  | Actinobacteria | 73.38 | <0.001 |  | 0.40 | 0.673 |  | 7.58 | <0.001 |
|  | Bacilli | 19.49 | <0.001 |  | 1.84 | 0.181 |  | 1.80 | 0.144 |
| Day 120 | Alphaproteobacteria | 2.86 | 0.059 |  | 0.28 | 0.751 |  | 0.61 | 0.715 |
|  | Gammaproteobacteria | 11.26 | <0.001 |  | 8.19 | 0.002 |  | 0.99 | 0.456 |
|  | Bacteroidia | 1.85 | 0.167 |  | 5.12 | 0.015 |  | 1.21 | 0.336 |
|  | Acidimicrobiia | 9.44 | <0.001 |  | 0.29 | 0.746 |  | 0.97 | 0.466 |
|  | Actinobacteria | 13.13 | <0.001 |  | 0.27 | 0.767 |  | 2.79 | 0.035 |
|  | Bacilli | 53.33 | <0.001 |  | 0.09 | 0.916 |  | 0.43 | 0.850 |
|  | Clostridia | 8.84 | <0.001 |  | 9.72 | <0.001 |  | 5.71 | <0.001 |
|  | Anaerolineae | 22.63 | <0.001 |  | 8.24 | 0.002 |  | 0.77 | 0.599 |
|  | Deltaproteobacteria | 2.87 | 0.059 |  | 1.14 | 0.336 |  | 2.46 | 0.056 |
|  | Subgroup_6 | 11.65 | <0.001 |  | 18.29 | <0.001 |  | 2.33 | 0.067 |

Note: **P* < 0.05, ***P* < 0.01.


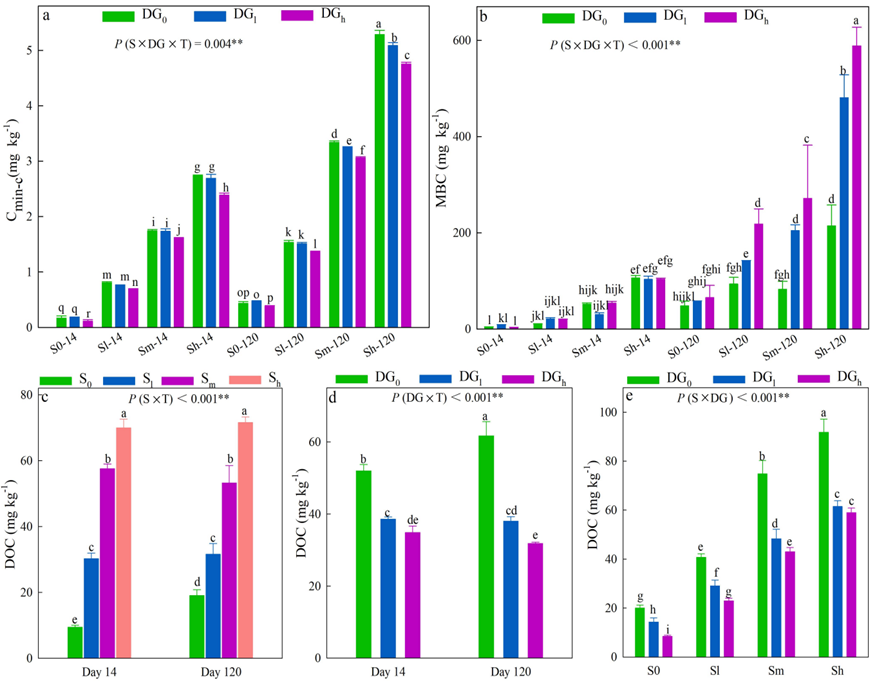


**FIGURE S1** Soil C_min-c_, MBC, and DOC as influenced by different levels of with straw and desulfurization gypsum treatment on days 14 and 120. Different lowercase letters indicate significant differences at the 5% level. The character shows the summary of a rmANOVA with “*p*” values (S, straw level; T, sampling time, DG, Desulfurization gypsum level; and S×T, DG×T, S×DG, S×DG×T, which indicate different interactions). C_min-c_, cumulative CO_2_ emission; DOC, dissolved organic carbon; MBC, microbial biomass carbon; rmANOVA, repeated measures analysis of variance; SMBC, microbial biomass carbon.


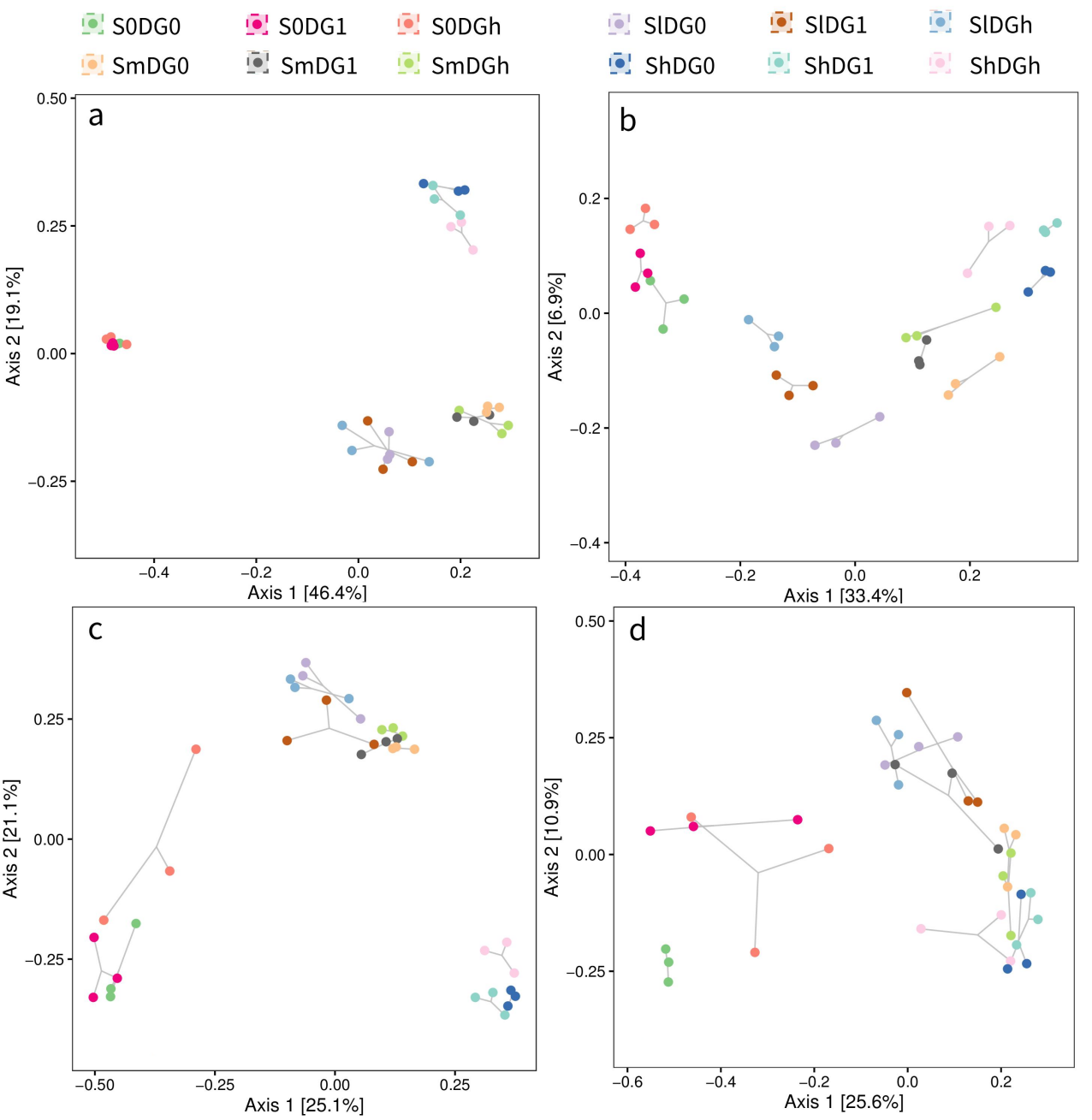


*R^2^*=0.817, *P*＜0.001**

*R^2^*=0.590, *P*＜0.001**

*R^2^*=0.627, *P*＜0.001**

*R^2^*=0.712, *P*＜0.001**

*R^2^*=0.627, *P*＜0.001**

*R^2^*=0.712, *P*＜0.001**

**FIGURE S2** A principal coordinates analysis (PCoA) plot depicts the Bray-Curtis distance of the bacterial communities on day 14 (a), and day 120 (b) and the fungal communities on day 14 (c) and day 120 (d) in 36 soil samples under the 12 treatment regimes.


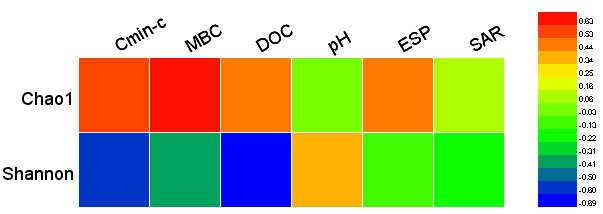

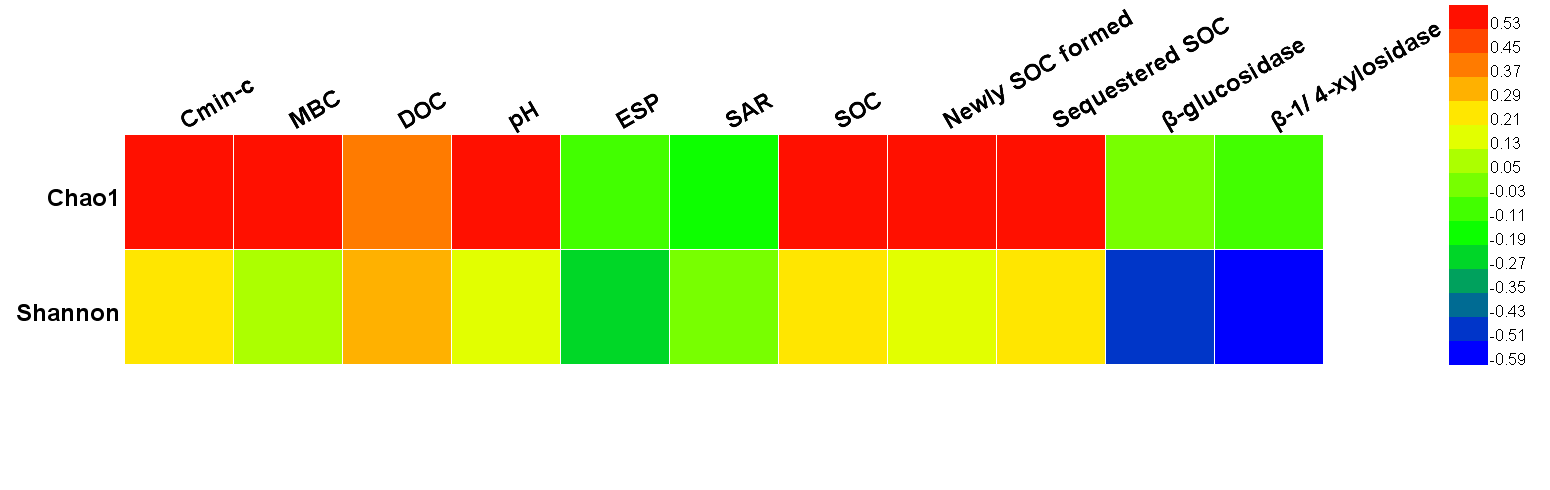


Day 14

Day 120

*

**

*

**

*

*

*

*

*

**FIGURE S3** Spearman’s rank correlation coefficients between soil biochemical properties and bacterial diversity on days 14 and 120 of the incubation. ***P*<0.01, **P*<0.05.

**


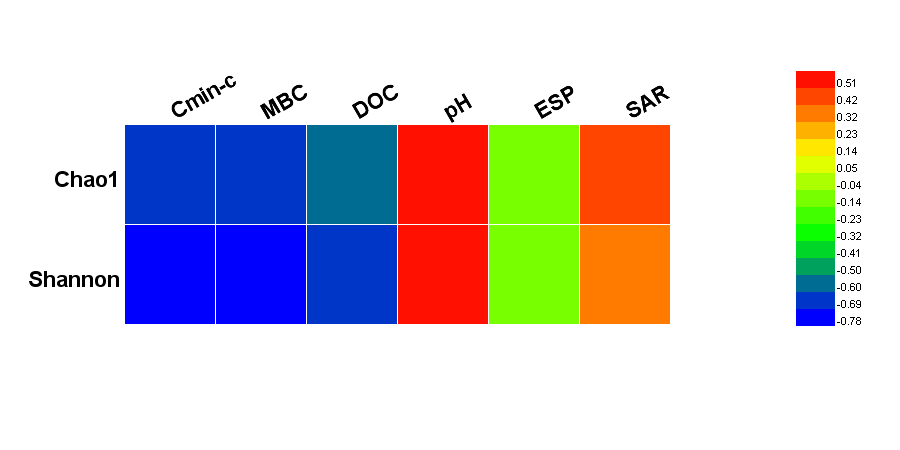

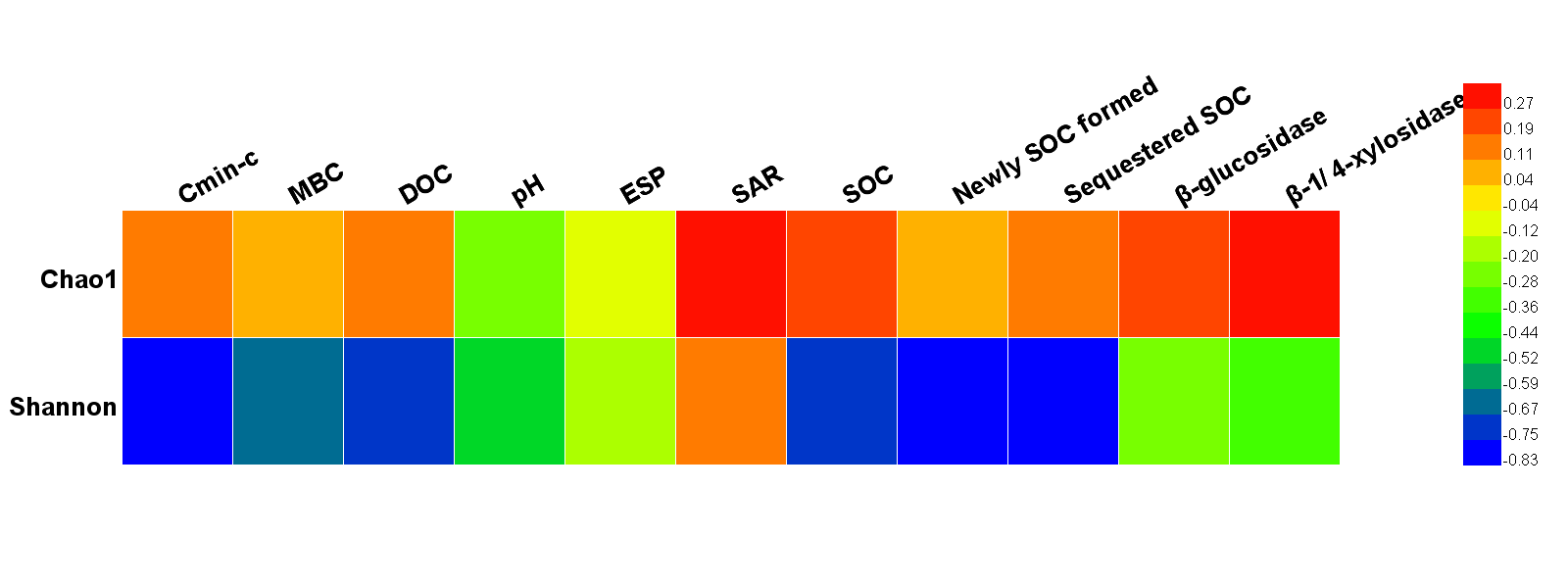


Day 14

Day 120

*

*

*

**

*

**

*

*

**

*

**

*

**

*

*

*

**FIGURE S4** Spearman’s rank correlation coefficients between soil biochemical properties and fungal diversity on days 14 and 120 of the incubation. ***P*<0.01, **P*<0.05.


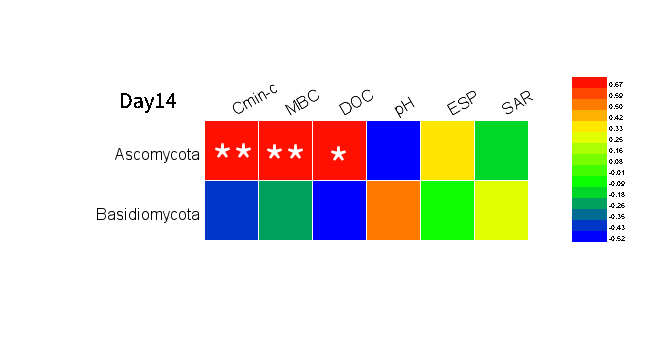

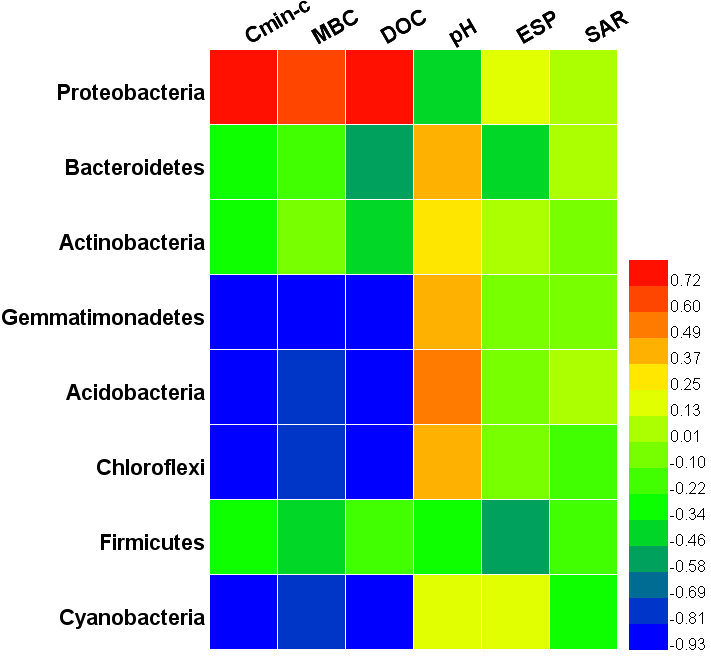

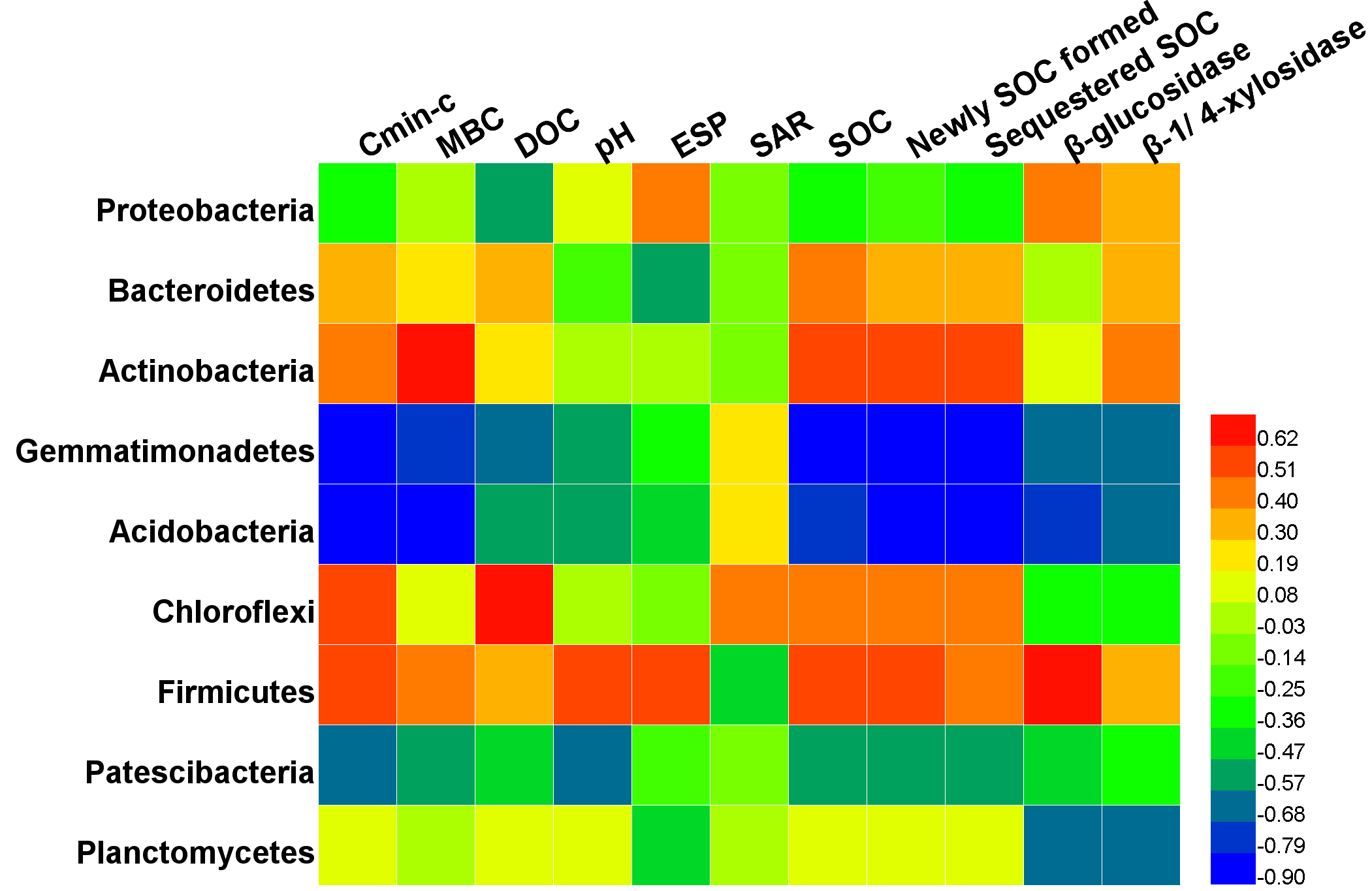


Day 14

Day 120

**

*

**

*

**

*

**

*

**

*

**

*

**

*

**

*

**

*

**

*

**

*

**

*

**

*

**

*

*

*

*

*

*

*

*

*

*

*

*

*

*

*

*

*

*

*

*

*

*

*

*

*

*

*

*

*

*

*

*

*

*

*

**

*

**

*

**

*

**

*

**

*

**

*

**

*

**

*

**

*

**

*

**

*

**

*

**

*

**FIGURE S5** Spearman’s rank correlation coefficients between soil biochemical properties and the relative abundance of the dominant bacterial phyla on days 14 and 120 of the incubation. ***P*<0.01, **P*<0.05.


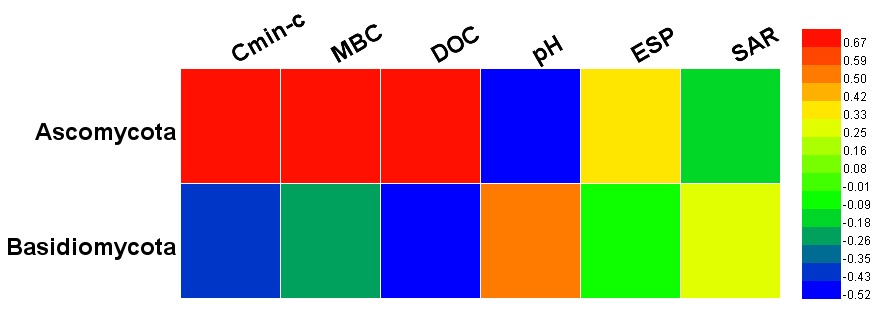

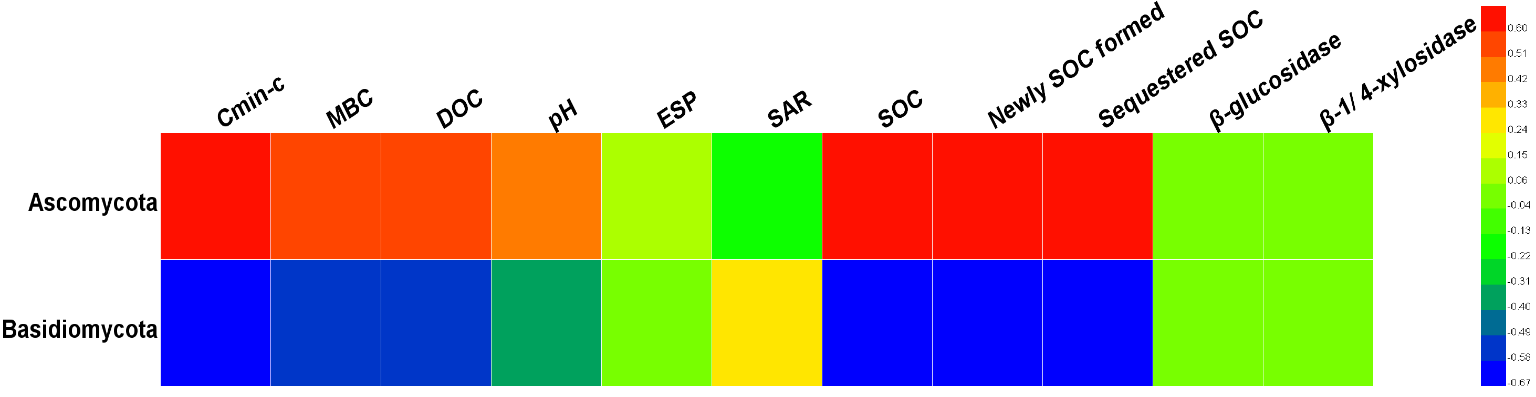


Day 14

Day 120

**

*

**

*

**

*

*

*

*

*

*

*

*

*

*

*

*

*

*

*

*

*

*

*

**FIGURE S6** Spearman’s rank correlation coefficients between soil biochemical properties and the relative abundance of the dominant fungal phyla on days 14 and 120 of the incubation. ***P*<0.01, **P*<0.05.


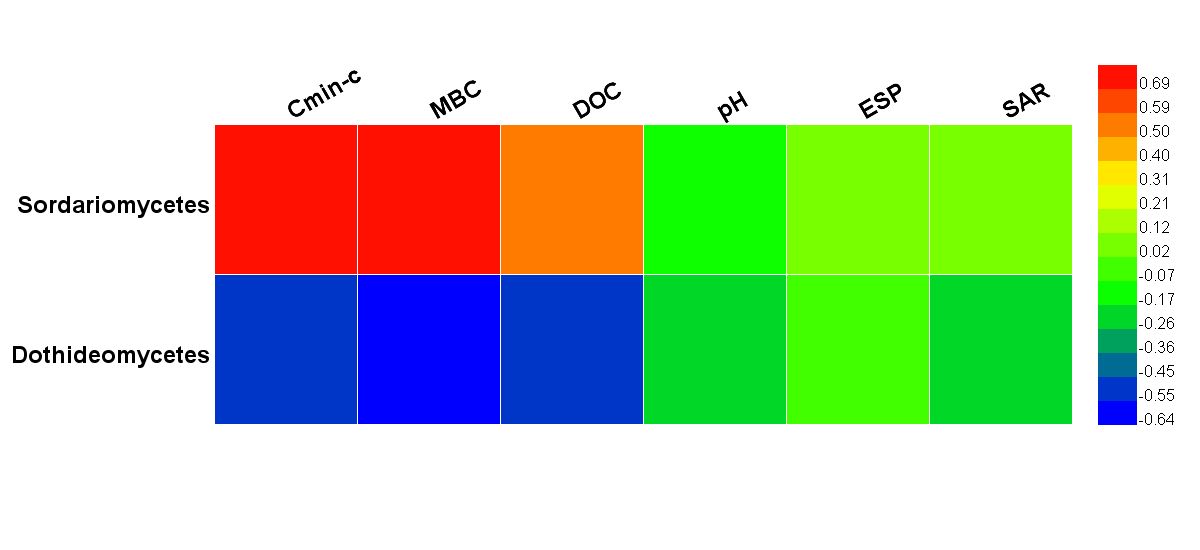

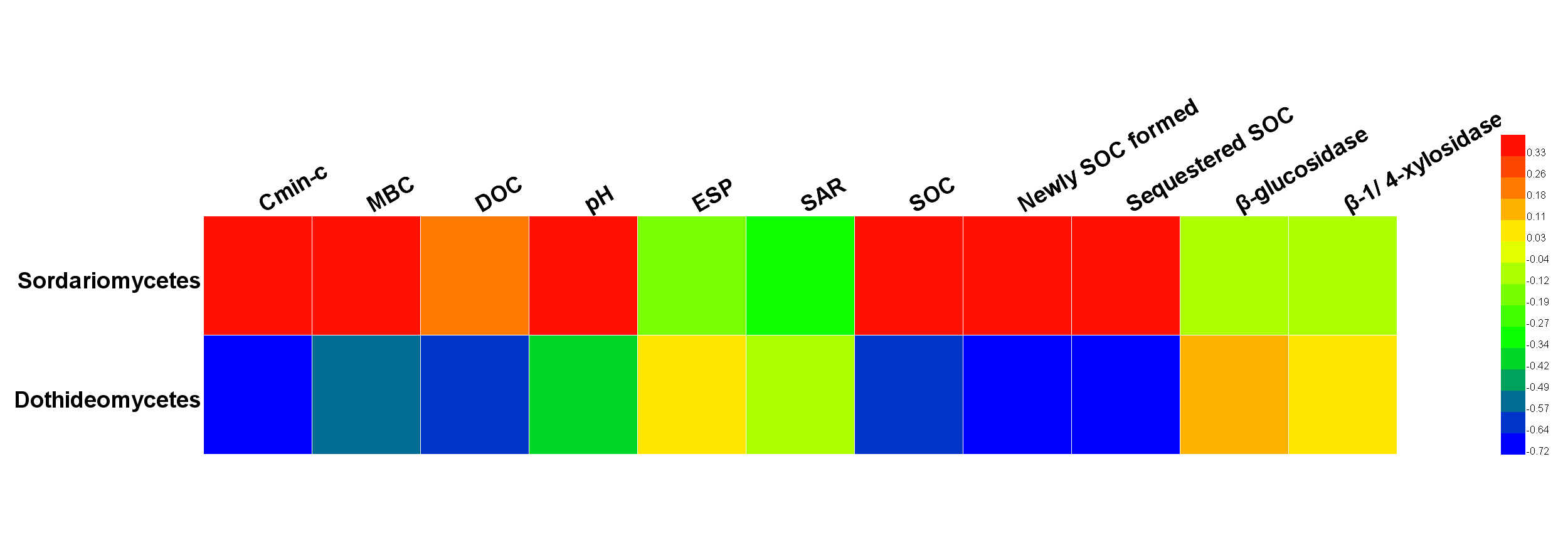


Day 14

Day 120

**

*

**

*

**

*

**

*

*

*

*

*

*

*

*

*

*

*

**FIGURE S7** Spearman’s rank correlation coefficients between soil biochemical properties and the relative abundance of the dominant fungal class on days 14 and 120 of the incubation. ***P*<0.01, **P*<0.05.


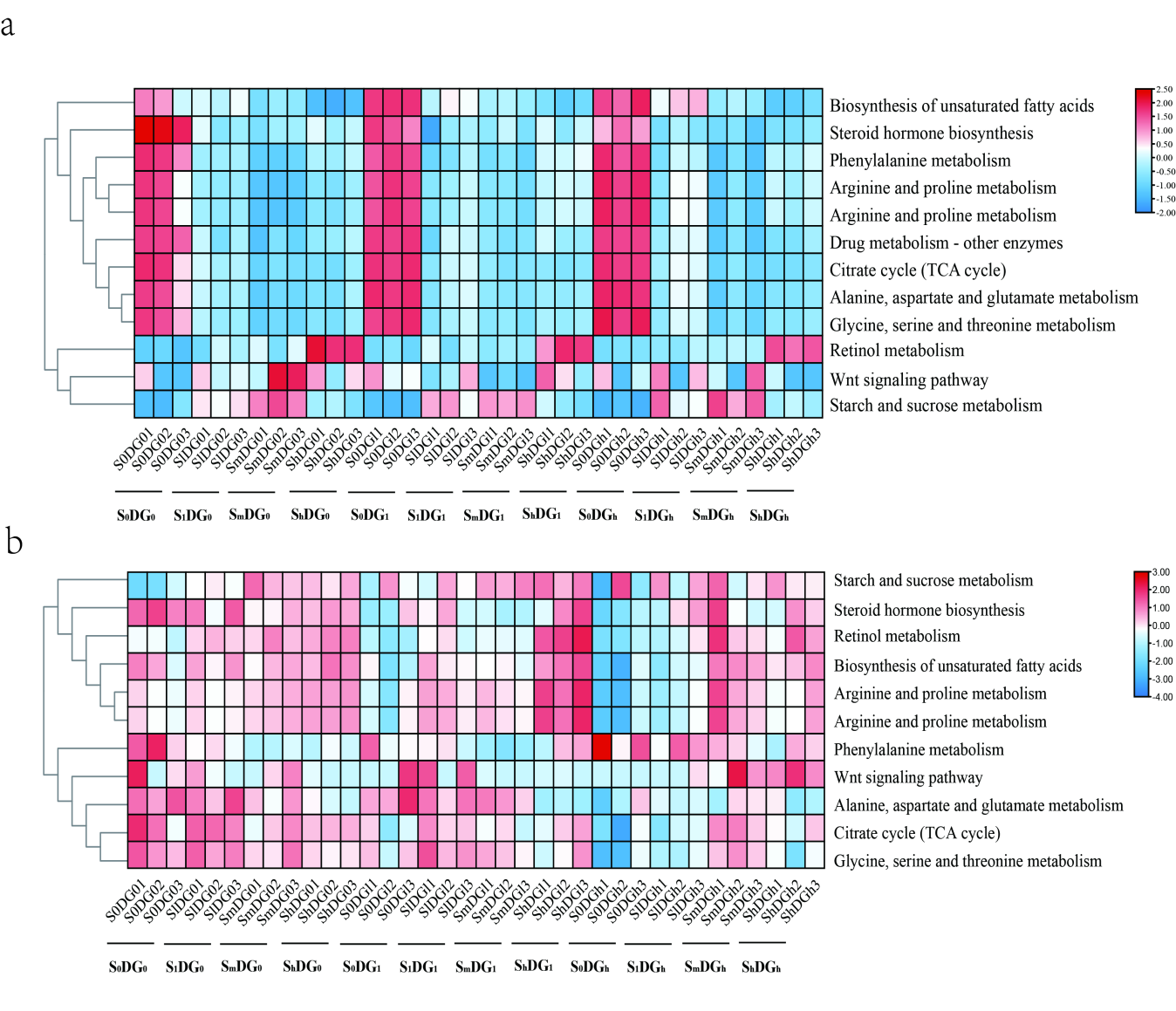


**FIGURE S8** Abundance of soil bacterial community functional groups predicted with the picrust 2 tool relative to treatment (a) on day 14 and (b) on day 120.
